# A mosaic of conserved and novel modes of gene expression and morphogenesis in mesoderm and muscle formation of a larval bivalve

**DOI:** 10.1007/s13127-022-00569-5

**Published:** 2022-07-07

**Authors:** Stephan M. Schulreich, David A. Salamanca-Díaz, Elisabeth Zieger, Andrew D. Calcino, Andreas Wanninger

**Affiliations:** Unit for Integrative Zoology, Department of Evolutionary Biology, University of Vienna, Djerassiplatz 1, 1030 Vienna, Austria

**Keywords:** Evodevo, Development, Evolution, Mollusca, Novelty, Myogenesis

## Abstract

The mesoderm gives rise to several key morphological features of bilaterian animals including endoskeletal elements and the musculature. A number of regulatory genes involved in mesoderm and/or muscle formation (e.g., *Brachyury* (*Bra*), *even-skipped* (*eve*), *Mox*, *myosin II heavy chain* (*mhc*)) have been identified chiefly from chordates and the ecdysozoans *Drosophila* and *Caenorhabditis elegans*, but data for non-model protostomes, especially those belonging to the ecdysozoan sister clade, Lophotrochozoa (e.g., flatworms, annelids, mollusks), are only beginning to emerge. Within the lophotrochozoans, Mollusca constitutes the most speciose and diverse phylum. Interestingly, however, information on the morphological and molecular underpinnings of key ontogenetic processes such as mesoderm formation and myogenesis remains scarce even for prominent molluscan sublineages such as the bivalves. Here, we investigated myogenesis and developmental expression of *Bra*, *eve*, *Mox*, and *mhc* in the quagga mussel *Dreissena rostriformis*, an invasive freshwater bivalve and an emerging model in invertebrate evodevo. We found that all four genes are expressed during mesoderm formation, but some show additional, individual sites of expression during ontogeny. While *Mox* and *mhc* are involved in early myogenesis, *eve* is also expressed in the embryonic shell field and *Bra* is additionally present in the foregut. Comparative analysis suggests that *Mox* has an ancestral role in mesoderm and possibly muscle formation in bilaterians, while *Bra* and *eve* are conserved regulators of mesoderm development of nephrozoans (protostomes and deuterostomes). The fully developed *Dreissena* veliger larva shows a highly complex muscular architecture, supporting a muscular ground pattern of autobranch bivalve larvae that includes at least a velum muscle ring, three or four pairs of velum retractors, one or two pairs of larval retractors, two pairs of foot retractors, a pedal plexus, possibly two pairs of mantle retractors, and the muscles of the pallial line, as well as an anterior and a posterior adductor. As is typical for their molluscan kin, remodelling and loss of prominent larval features such as the velum musculature and various retractor systems appear to be also common in bivalves.

**Supplementary information:**

The online version contains supplementary material available at 10.1007/s13127-022-00569-5.

## Introduction

Bilaterian animals have three germ layers, the ectoderm, the endoderm, and the mesoderm. The mesoderm originates during gastrulation and forms a variety of derivatives, including connective tissue and the musculature. Gene expression during mesoderm formation and/or myogenesis has been studied in most bilaterians such as acoelomorphs, deuterostomes such as chordates, hemichordates, and echinoderms, as well as in ecdysozoan and lophotrochozoan protostomes (e.g., brachiopods, ectoprocts, phoronids, and annelids; Candia & Wright, 1995; Furlong et al., 2001; Minguillón & Garcia-Fernàndez, 2002; Pocock et al., 2004; Lowe et al., 2006; Andrikou et al., 2013; Chiodin et al., 2013; Andrikou & Arnone, 2015; Passamaneck et al., 2015; Erkenbrack, 2016; Kozin et al., 2016; Martín-Durán et al., 2017; Vellutini et al., 2017; Andrikou & Hejnol, 2021). Nevertheless, a large gap of knowledge exists for one of the most morphologically diverse lophotrochozoan phyla, Mollusca, for which only few species have been investigated in some detail (e.g., the gastropod *Crepidula fornicata* and the bivalve *Saccostrea kegaki*; Kakoi et al., 2008; Perry et al., 2015).

Developmental genes with a widely conserved expression during bilaterian mesoderm formation are manifold and include *Brachyury* (*Bra*), *caudal* (*cdx*), *dachshund* (*dachs*), *even-skipped* (*eve*), *eyes absent* (*eya*), *forkhead A* (*foxA*), *forkhead C* (*foxC*), *forkhead D* (*foxD*), *forkhead F* (*foxF*), *gata4/5/6*, *myocyte enhancer factor-2* (*mef2*), *Mox*, *myosin II heavy chain* (*mhc*), *myoblast determination protein 1* (*myoD*), *neurokinin 1* (*nk1*), *paraxis*, *sine oculis* (*six1/2*), *snail*, *tropomyosin* (*tm*), *twist* (*twi*), and *vasa* (*vas*) (Andrikou & Hejnol, 2021; Martín-Durán et al., 2017; Passamaneck et al., 2015; Sebé-Pedrós & Ruiz-Trillo, 2017; Zhang & Bernstein, 2001). The homeobox gene *Mox* (a homolog of *Meox*, *Gax*, and *buttonless*) appears to have an additional role in myogenesis in some lophotrochozoans and chordates (Kozin et al., 2016; Passamaneck et al., 2015; Satou & Imai, 2015). *Eve* (a homolog of *Evx*, *Xhox3*, and *vab-7*) is closely related to *Mox* and acts as a pair-rule gene during arthropod segmentation (Copf et al., 2003; Damen et al., 2000; Janssen et al., 2011; Patel et al., 1994). It is also involved in mesoderm development and/or myogenesis in cephalochordates, vertebrates, and ecdysozoans, as well as in vertebrate limb formation (Ruiz et al., 1989; Patel et al., 1992; Ahringer, 1996; Hérault et al., 1996; Sordino et al., 1996; Ferrier et al., 2001; Fujioka et al., 2005). *Bra* is expressed in the mesoderm of a number of protostomes and deuterostomes including annelids, brachiopods, priapulids, and arthropods (Kozin et al., 2016; Kusch & Reuter, 1999; Martín-Durán et al., 2017; Peter & Davidson, 2011; Peterson et al., 1999; Sebé-Pedrós & Ruiz-Trillo, 2017). For mollusks, no mesodermal expression of *Bra* was found in the gastropod *Haliotis asinina*, whereas in another marine snail, *Patella vulgata*, *Bra* is transiently expressed in the 4d cell that gives rise to the future endomesoderm (Koop et al., 2007; Lartillot et al., 2002). In the gastropod *Crepidula fornicata*, *Bra* is involved in mesoderm formation, while in the bivalves *Crassostrea gigas* and *Saccostrea kegaki*, the data are somewhat inconclusive as to whether or not *Bra* is expressed during mesoderm formation (Kin et al., 2009; Perry et al., 2015; Tan et al., 2017). During metazoan myogenesis, a number of genes and their respective proteins are commonly expressed, including those of the myosin family (Burgess, 2005; Thompson & Langford, 2002). Of these, *myosin II heavy chain* (*mhc*) appears to have a particularly conserved role in muscle formation and is consistently expressed from the earliest stages of myogenesis onwards in a number of phyla (Kobayashi et al., 1998; Zhang & Bernstein, 2001; Renfer et al., 2010; Andrikou et al., 2013).

Although larval myoanatomy has been described in several invertebrate taxa including mollusks, very few details are available on the ontogenetic sequence that gives rise to the highly intricate musculature of larval and adult bivalves, the second largest class-level molluscan taxon after the gastropods (Audino et al., 2015; Li et al., 2019; Sun et al., 2019; Wurzinger-Mayer et al., 2014). These studies showed that bivalve larvae typically exhibit a velum muscle ring as well as various retractor systems that degenerate prior to or at metamorphosis. The muscles of the pallial line, the mantle retractors, the adductor system, as well as the foot retractors together with the plexus-like foot musculature, are common features of adult bivalves that develop in the larva and are retained after metamorphosis (Audino et al., 2015; Cragg, 2016; Li et al., 2019; Sun et al., 2020; Wurzinger-Mayer et al., 2014).

The invasive quagga mussel *Dreissena rostriformis* (Deshayes, 1838) shows an indirect lifecycle with a trochophore and a subsequent veliger larva, and is an emerging model system in evolutionary developmental biology (Calcino et al., 2019; Salamanca-Díaz et al., 2021). In order to assess whether common regulators of bilaterian mesoderm and muscle formation are also involved in bivalve ontogeny, we investigated the expression of *Brachyury*, *even-skipped*, *Mox*, and *myosin II heavy chain* during *D. rostriformis* development. In addition, we provide a detailed account of myogenesis in this model bivalve in order to contribute to the reconstruction of the myoanatomical ground pattern of bivalve larvae.

## Materials and methods

### Animal collection, spawning, and fixation

Adult quagga mussels were collected in the Danube River in Vienna, Austria (Georg-Danzer-Steg, 48°14ʹ45.7ʺN 16°23ʹ38.4ʺE), in May 2018. Mussels were kept in a 45 L aquarium in an incubator at 18 °C in Danube water with a weekly water change. Prior to spawning, adult mussels were cleaned with a brush under running tap water. The specimens were washed in a 100 mL beaker with 2 µm filtered Danube water (FDW) containing 0.1% sodium hypochlorite (#09,951,780, DanKlorix, Hamburg, Germany) for 5 min. To induce spawning, the mussels were placed in a fresh 100 mL beaker with FDW containing 10^−3^ M serotonin hydrochloride (#H9523, Sigma-Aldrich, St. Louis, MO, USA) for 20 min at room temperature (RT). After gamete release, the eggs of each female were mixed with two to three drops of concentrated sperm and transferred to a 200 mL container with fresh FDW and incubated for 15 min. This was followed by three washes in FDW to remove excess sperm. When the animals had reached the trochophore stage, the larvae of each female were transferred to a fresh container with 2 L FDW with aeration and a magnetic stirrer and were kept at 18 °C. The FDW was exchanged every 2 days, and when the veliger stage was reached, the larvae were additionally fed one to two drops of an *Isochrysis* concentrate after the water change (Plankton-Welt, Hamburg, Germany).

Prior to fixation, crystalline cocaine was added to veliger larvae at a final concentration of 30 µg/mL (#609,020,011, Gatt-Koller, Absam, Austria) to avoid retraction into the shell. Developmental stages (gastrula, trochophore larva, early D-shaped veliger larva, late veliger larva) were fixed in 4% ice-cold paraformaldehyde (PFA) (#158,127, Sigma-Aldrich) in 0.1 M phosphate buffer saline (PBS) for 1 h. For in situ hybridization, samples were washed 2 × 10 min in 100% methanol and stored at − 20 °C. For immunofluorescence and actin staining, larvae were washed 3 × 10 min in PBS containing 0.1% NaN_3_ (#71,289, Sigma-Aldrich) and stored at 4 °C.

### Fluorescence staining

*Dreissena rostriformis* samples were washed 3 × 10 min in PBS, followed by decalcification for 1 h in 50 mM EGTA (#E3889, Sigma-Aldrich) in PBT (1 × PBS, 0.1% Tween 20; #9127.1, Carl Roth, Karlsruhe, Germany) and 2 × 10 min washes in PBT at RT. Unspecific binding sites were blocked for 1 h in PBT with 3% normal swine serum (#014–000-121, Jackson ImmunoResearch, West Grove, PA, USA). Subsequently, samples were incubated in the primary antibodies (dilution 1:900, anti-acetylated α-tubulin, #T6793, Sigma-Aldrich) in the block solution overnight at RT. All specimens were washed 5 × 15 min in PBT and incubated in secondary antibodies (dilution 1:900, goat anti-mouse, Alexa Fluor 633, #A21050, Invitrogen, Carlsbad, CA, USA) with DAPI (dilution 1:400, 4ʹ,6-diamidino-2-phenylindole, #D1306, Invitrogen) added to visualize cell nuclei and Alexa Fluor 488 phalloidin (dilution 1:40, #A12379, Invitrogen) for actin labelling in PBT for 24 h at 4 °C in the dark. All samples were washed 5 × 15 min in PBT, followed by two washing steps in PBS for 10 min each. Stained specimens were mounted on glass slides with Fluoromount-G (#0100–01, SouthernBiotech, Birmingham, AL, USA). The samples were stored at 4 °C in the dark for a few days prior to the analyses. Samples were analysed with a Leica SP5 II confocal laser scanning microscope with the software LAS AF (v. 2.6.3.8173) (both Leica Microsystems, Wetzlar, Germany). ImageJ2 (Rasband, W.S., ImageJ, US National Institutes of Health, Bethesda, MD, USA, https://imagej.nih.gov/ij/, 1997–2018) and Imaris × 64 (v. 7.3.1) (Bitplane, Zurich, Switzerland) were used to analyse the image stacks, and Inkscape (v. 0.92.4; https://inkscape.org/) was used to create the schematic drawings.

### Bioinformatic analysis

Most candidate orthologs of the genes of interest (*myosin II heavy chain*, *Mox*, *even-skipped*, and *Brachyury*) and corresponding outgroups were retrieved from the NCBI nr database (https://www.ncbi.nlm.nih.gov) and confirmed with reciprocal blast searches (Supplemental Tables 1, 2, and 3). *Dreissena rostriformis* sequences were subsequently obtained by BLASTp (v. 2.8.1 +) against the translated transcriptome using these candidate sequences as queries (Calcino et al., 2019). Additionally, a few orthologs were downloaded from the Ensembl Metazoa database (Supplemental Tables 1 and 3). Orthologs containing either the myosin head domain or the T-box domain from the bivalve *Crassostrea gigas* and the cnidarian *Nematostella vectensis* were obtained by using hmmscan (Eddy, 1995) with the corresponding PFam (v. 32.0) hmm files (PF00063.21, PF00907.22) against the respective Ensembl genomes (Howe et al., 2020; Hinxton, UK, https://metazoa.ensembl.org/index.html). All orthologs of the genes of interest of *Acanthochitona fascicularis* were identified by blast hits against the transcriptome (De Oliveira et al., 2016; here assigned to *A. crinita*) (https://zoology.univie.ac.at/research/open-data/) using hmmscan (Eddy, 1995).

For the *myosin II heavy chain* phylogeny, myosin families that are commonly known from metazoans were included (Thompson & Langford, 2002). All selected myosin families contain a myosin head domain and because *myosin I* is considered to be the earliest branching family, it was used as the outgroup for the phylogeny (Foth et al., 2006). For the *even-skipped* and *Mox* phylogenies, several *Hox* gene families were used as outgroup (Minguillón & Garcia-Fernàndez, 2003; Ryan et al., 2007). For the *Brachyury* phylogeny, all metazoan-specific T-box families were included. *Brachyury* is an early branching family of T-box proteins and so was set as an outgroup to the remaining T-box families (Sebé-Pedrós & Ruiz-Trillo, 2017; Sebé-Pedrós et al., 2013).

Multiple sequence alignments were performed using MAFFT (v. 7.427) (Katoh et al., 2002), trimming was performed with BMGE (v. 1.12) (Criscuolo & Gribaldo, 2010), visualisation was performed with AliView (v. 1.0.0.0) (Larsson, 2014), and editing was performed with Jalview (v. 2.11.0) (Waterhouse et al., 2009). Appropriate amino acid substitution models were determined using ProtTest (v. 2.1) (Abascal et al., 2005). These were LG (Le & Gascuel, 2008) for *Brachyury* and *myosin II heavy chain* and JTT (Jones et al., 1992) for e*ven-skipped* and *Mox*. The phylogenetic (maximum likelihood) trees were computed using PHYML (v. 3.1) (Guindon & Gascuel, 2003) with a bootstrap value of 100. Visualisation of phylogenetic trees was performed with FigTree (v. 1.4.4) (http://tree.bio.ed.ac.uk/software/figtree/). For in situ hybridization probe production, specific primers for each gene under investigation were designed manually (Supplemental Table 4) and synthesised by Microsynth Austria GmbH (Vienna, Austria). Reading frames and orientation of the transcriptomic templates were verified with the ExPASy translate tool (Artimo et al., 2012; https://web.expasy.org/translate/) and melting temperatures of the designed primers were checked with the Promega Oligo Calculator tool (Rychlik & Rhoads, 1989; https://at.promega.com/resources/tools/biomath/tm-calculator/; 500 nM primer concentration, 5 × green or colourless Go Taq Reaction Buffer). For the self-complementary check, the Northwestern biotool OligoCalc tool (Kibbe, 2007; http://biotools.nubic.northwestern.edu/OligoCalc.html) was used. The primers were diluted to yield a working concentration of 10 µM and stored at − 20 °C. The nucleotide sequences and insert length of each primer pair are listed in Supplemental Table 4. Relative gene expression values (tpm values) were retrieved for all four genes of interest using stage-specific transcriptomes of *D. rostriformis* (Calcino et al., 2019).

### RNA extraction, gene cloning, and probe synthesis

Different developmental stages (1, 2, 4, 6, 8, 12, 15, 18, 21, 24, 28, 48, 70 h post fertilisation; hpf) were transferred to RNAlater (#76,106, Qiagen, Venlo, Netherlands) and stored at 4 °C. For total RNA extraction from pooled stages, the RNeasy Mini Kit (#74,104, Qiagen) with the QIAshredder homogeniser (#79,654, Qiagen) was used according to the manufacturer’s instructions. RNA samples were diluted 1:10 with DEPC (diethylpyrocarbonate)–treated water, quantified by a spectrophotometer (Nanodrop 2000c, Thermo Fisher Scientific), and stored at −80 °C. For cDNA synthesis, total RNA was denatured for 15 min at 65 °C and placed on ice. Subsequently, the 1st Strand cDNA Synthesis Kit for RT-PCR (#11 483 188 001, Roche, Basel, Switzerland) was used with Oligo-p(dt)_15_ primers. The obtained cDNA was diluted 1:5 with DEPC-treated water and stored at −20 °C.

PCRs (cDNA-, plasmid-, colony-PCR) were performed using Go Taq Flexi DNA Polymerase (0.025 U/µl, #M780B, Promega, Madison, WI, USA), 1 × Go Taq Flexi Buffer (#M890A, Promega), PCR nucleotide mix (0.8 mM, #C1145, Promega), 1.25 mM MgCl_2_ (#A351H, Promega), and nuclease-free water (#R0581, Thermo Fisher Scientific, Waltham, MA, USA). To amplify *Brachyury*, *Mox*, *even-skipped*, and *myosin II heavy chain*, gene-specific primers (Supplemental Table 4) and cDNA were added to the PCR mixture. The PCR products were checked on a 1% agarose gel (#2267.4, Carl Roth) in TAE buffer (#CL86.1, Carl Roth). Bands corresponding to the expected nucleotide sequence length were excised and the DNA was extracted using the QIAquick Gel Extraction Kit (#28,706, Qiagen). The extracted DNA (insert) was stored at −20 °C. Ligation of the insert into a plasmid and transformation of the plasmid into *E. coli* JM109 Competent Cells were done using the pGEM-T Easy Vector System II (#A1380, Promega) according to the manufacturer’s instructions. White-blue screening of transformed bacteria was performed on LB agar plates (35 mg/mL, #965.1, Carl Roth) with 0.1% ampicillin (#A9518, Sigma-Aldrich). Successful transformation of the desired insert was confirmed by colony PCR, using M13 primers (10 µM, Microsynth, Balgach, Switzerland). Transformed bacteria were grown in 5 mL LB medium (#X964.1, Carl Roth) containing ampicillin (100 µg/mL, #A9518, Sigma-Aldrich) overnight at 37 °C with agitation (180 RPM). Plasmids were purified using the QIAprep Spin Miniprep Kit (#27,106, Qiagen), quantified (Nanodrop 2000c, Thermo Fisher Scientific), and sequenced (Microsynth, Vienna, Austria).

Inserts corresponding to genes of interest were amplified through plasmid PCR using M13 primers. PCR products were checked by gel electrophoresis and stored at 4 °C. For the synthesis of sense and anti-sense riboprobes, PCR products (100–200 ng) were incubated with RNase-free water (#R0581, Thermo Fisher Scientific), 1 × transcription buffer (#11,465,384,001, Roche), 10 µM dithiothreitol (DTT, #D9779, Sigma-Aldrich), 1 × DIG RNA Labelling Mix (#11,277,073,910, Roche), 0.1 U Protector RNase Inhibitor (#03,335,402,001, Roche), 50 U SP6 RNA polymerase (#10,810,274,001, Roche), or 50 U T7 RNA polymerase (#10,881,767,001, Roche) in a thermocycler (37 °C, lid 60 °C) for 2 h. Afterwards, 1 µL DNase I (recombinant, RNase-free, #04,716,728,001, Roche) was added and samples were incubated for another 15 min at 37 °C to remove template DNA. DIG-Probes were purified via ProbeQuant™ G-50 Micro Columns (#GE28-9034–08, GE Healthcare, Chicago, IL, USA).

Riboprobes were precipitated by adding 5 µL 4 M LiCl (#L7026, Sigma-Aldrich) and 120 µL 100% EtOH (#20,821, VWR Chemicals, Radnor, PA, USA) and by incubating overnight at −20 °C. Next, riboprobes were centrifuged at 14,000 RPM for 15 min at 4 °C and the obtained pellets were washed twice with 70% EtOH. Pellets were dried for 15 min at RT and dissolved in 20 µL RNase-free water (#R0581, Thermo Fisher Scientific). All RNA probes were quantified by a spectrophotometer, checked by gel electrophoresis, and stored at −80 °C.

### Whole mount in situ hybridization (WMISH)

Prior to WMISH, the developmental transcript abundances of each target gene were checked using quantitative gene expression data (Supplemental Table 5) (Calcino et al., 2019). This was done in order to assess the relative expression levels of putative genes of interest and helped in choosing promising candidate genes as well as key developmental stages for in situ hybridization experiments. Full-length sequences of the riboprobes used for WMISH experiments are provided in Supplemental Table 6.

*Dreissena rostriformis* samples were rehydrated stepwise from 100% methanol to 0.1 M PBS (#1058.1, Carl Roth). All samples were decalcified for 1 h in PPE (4% PFA (#158,127, Sigma-Aldrich), 0.1 M PBS, 50 mM EGTA pH 8 (#E3889, Sigma-Aldrich)) and washed 3 × 5 min in PBT. Subsequently, the larvae were incubated in 30 µg/mL proteinase-K (#03,115,879,001, Roche) in PBS for 10 min at 37 °C. Specimens were washed 3 × 5 min in PBT, post-fixed in 4% PFA in PBS for 45 min, and washed again 3 × 5 min in PBT. Subsequently, the larvae were stepped into 100% hybridization buffer (50% formamide (#47,671, Sigma-Aldrich), 5 × SSC (#10,541, Carl Roth), 50–100 µg/mL heparin (#H3149, Sigma-Aldrich), 5 mM EDTA pH 8 (#20–158, Sigma-Aldrich), 1 × Denhardt’s (#D2532, Sigma-Aldrich), 100 µg/mL yeast tRNA (#R6750, Sigma-Aldrich), 0.1% Tween 20 (#9127.1, Carl Roth), 5% dextransulfat (#D8906, Sigma-Aldrich)). Pre-hybridization was carried out overnight at a gene-specific temperature (58.5 °C for *myosin II heavy chain* and 55 °C for *Mox*, *even-skipped*, and *Brachyury*). Each sense probe (negative control) and anti-sense probe was diluted at a concentration of 2 ng/µL in hybridization buffer and denatured for 10 min at 85 °C. After adding a riboprobe to the specimens, they were allowed to hybridize for 48–60 h at the abovementioned gene-specific temperatures.

The samples were washed 3 × 20 min in 4 × wash (50% formamide (#47,671, Sigma-Aldrich), 4 × SSC (#10,541, Carl Roth), 0.1% Tween 20 (#9127.1, Carl Roth)), followed by 2 × 20 min washes in 2 × wash (with 2 × instead of 4 × SSC) and another 2 × 15 min washes in 1 × wash (with 1 × SSC). Specimens were allowed to cool down to RT and washed 3 × 15 min in 1 × SSC (#10,541, Carl Roth) containing 0.1% Tween 20 (#9127.1, Carl Roth). Subsequently, all samples were stepped into 0.1 M MAB (100 mM maleic acid (#K304.1, Carl Roth), 150 mM NaCl (#6781.3, Carl Roth), 0.1% Tween 20 (#9127.1, Carl Roth)). Specimens were blocked for 3 h in blocking solution (2% blocking reagent (#11,096,176,001, Roche), 0.1 M MAB) at RT, followed by incubation in Anti-Digoxigenin-AP Fab fragments (#11,093,274,910, Roche) diluted 1:5000 in blocking solution overnight at 4 °C. Next, the samples were washed 3 × 20 min and 3 × 10 min in PBT. Prior to staining, the larvae were washed 2 × 5 min in AP buffer (1 × alkaline phosphatase, 1 M NaCl (#6781.3, Carl Roth), 200 mM Tris pH 9 (#4855.1, Carl Roth), 0.1% Tween 20 (#9127.1, Carl Roth)). For highly expressed genes (e.g., *myosin II heavy chain*), specimens were stained in colour reaction buffer (1 × AP buffer, 5 µL/mL NBT (nitroblue tetrazolium chloride, #11,383,213,001, Roche), 3.75 µL/mL BCIP (5-bromo-cloro-3-indolyl-phosphate, 4-toluidine salt (#11,383,221,001, Roche)) at 37 °C for 2–3 h. For lowly expressed genes, 7.5% polyvinyl alcohol (PVA) (#P1763, Sigma-Aldrich) was added to the colour reaction buffer and specimens were incubated at 37 °C for 4–13.5 h. In order to stop the reaction, the larvae were washed 2 × 5 min in PBT and post-fixed in 4% PFA for 1 h at 4 °C. Subsequently, the specimens were washed 3 × 5 min in PBT and 3 × 10 min in PBS at RT. All washing steps were done on a shaker at 130 RPM. Stained larvae were stored at 4 °C and the PBS was changed once a week. For the subsequent analyses, the samples were mounted on glass slides in 100% glycerol (#G5516, Sigma-Aldrich) and imaged using an Olympus BX53 light microscope equipped with an Olympus DP73 camera and the software cellSens Standard (v. 1.11) (Olympus Corporation, Shinjuku, Tokyo, Japan). Schematic drawings were created with Inkscape (v. 0.92.4).

## Results

### Phylogenetic analyses of genes of interest

All annotated genes of interest are summarized in Supplemental Table 7. For the *myosin II heavy chain* family, four candidates (*Dro-mhc_c1*, *c2*, *c3*, *c4*) were found in *Dreissena rostriformis*, which contain a specific glycine insertion (G) (Richards & Cavalier-Smith, 2005) at position 534 (Supplemental Fig. 1). The first three candidates (*Dro-mhc_c1*-*c3*) include a complete myosin N, myosin head, and myosin tail 1 domain. The fourth candidate (*Dro-mhc_c4*) contains a fragmented myosin head domain (Supplemental Table 7). Nine further candidates (two copies of *myosin I*, *myosin III*, *myosin V*, *myosin VI*, *myosin VII*, *myosin IX*, *myosin XV*, and *myosin XVIII*) with partially fragmented myosin head domains were found in *D. rostriformis* and nest within the corresponding myosin family (Supplemental Fig. 1a and Supplemental Table 7).

**Fig. 1:**
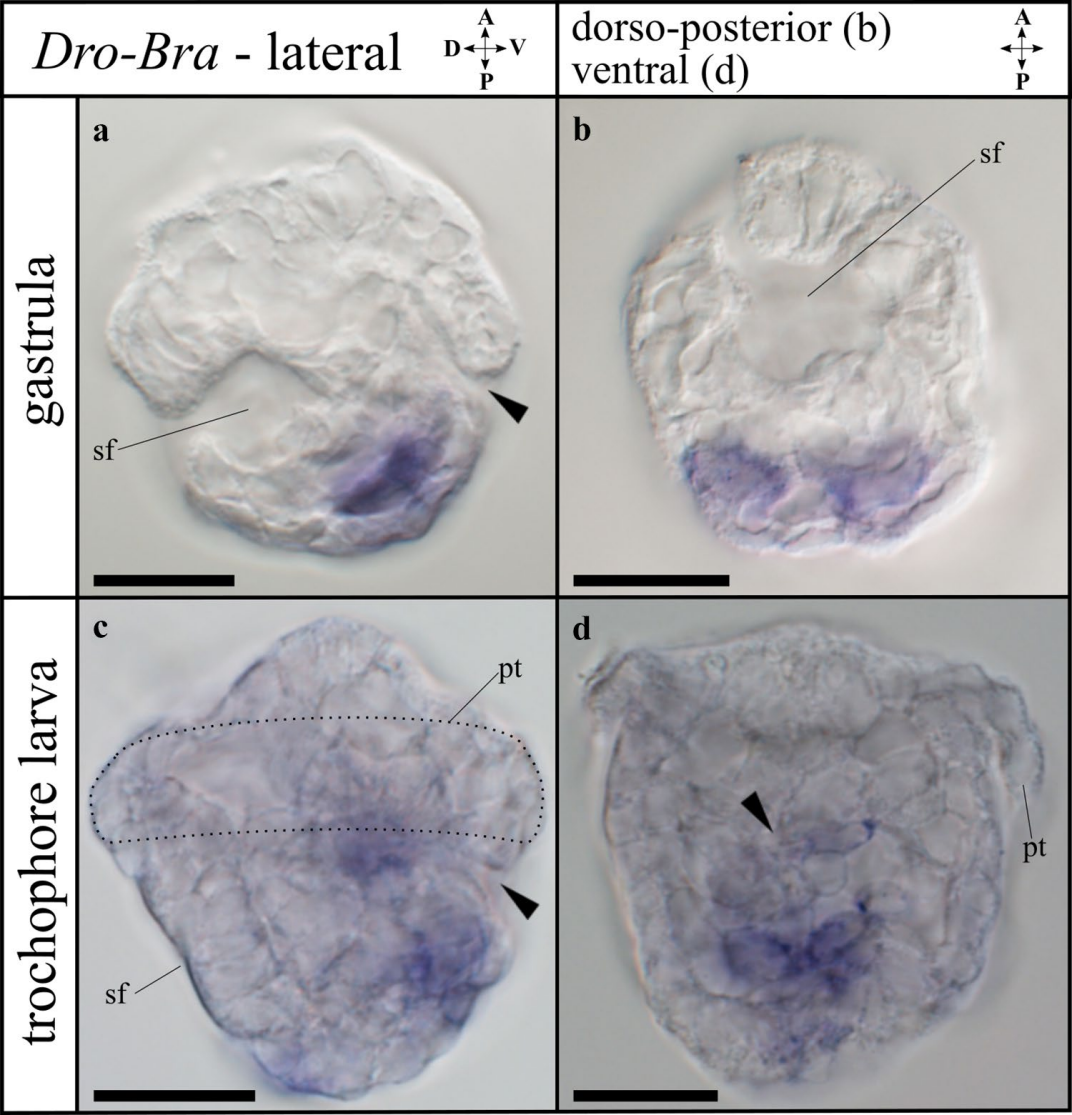
Expression of *Brachyury* (*Dro-Bra*) in *Dreissena rostriformis* gastrula and trochophore stages. Anterior is up. Black arrowheads indicate the blastopore/stomodaeum, sf marks the shell field, dotted line marks the region of the prototroch (pt). Scale bar equals 20 µm. **a** Lateral view of *Dro-Bra* in the ventral mesoderm. **b** Ventral view of mesodermal *Dro-Bra* expression. **c** *Dro-Bra* is expressed in the ventro-posterior mesoderm and in the developing foregut. **d** Mesodermal and endodermal expression of *Dro-Bra*, ventral view. The left and right lobes belong to the prototroch (pt). A, anterior; D, dorsal; P, posterior; V, ventral

A phylogenetic tree was constructed for the homeobox domain–containing *even-skipped* and *Mox* genes (Supplemental Fig. 2). A single *D. rostriformis even-skipped* (*Dro-eve*) ortholog was identified, which includes a proposed characteristic tyrosine (Y) at position 48 at the beginning of the homeobox domain (Supplemental Fig. 2a, b). Two *D. rostriformis Mox* (*Dro-Mox_c1* and *Dro-Mox_c2*) orthologs were found, which contain a putatively specific glutamic acid (E) insertion at position 48 at the beginning of the homeobox domain (Supplemental Fig. 2a, c). The third *Mox* candidate nests within the *Hox4* family and so is likely not a true *Mox* gene (Supplemental Fig. 2a).

**Fig. 2:**
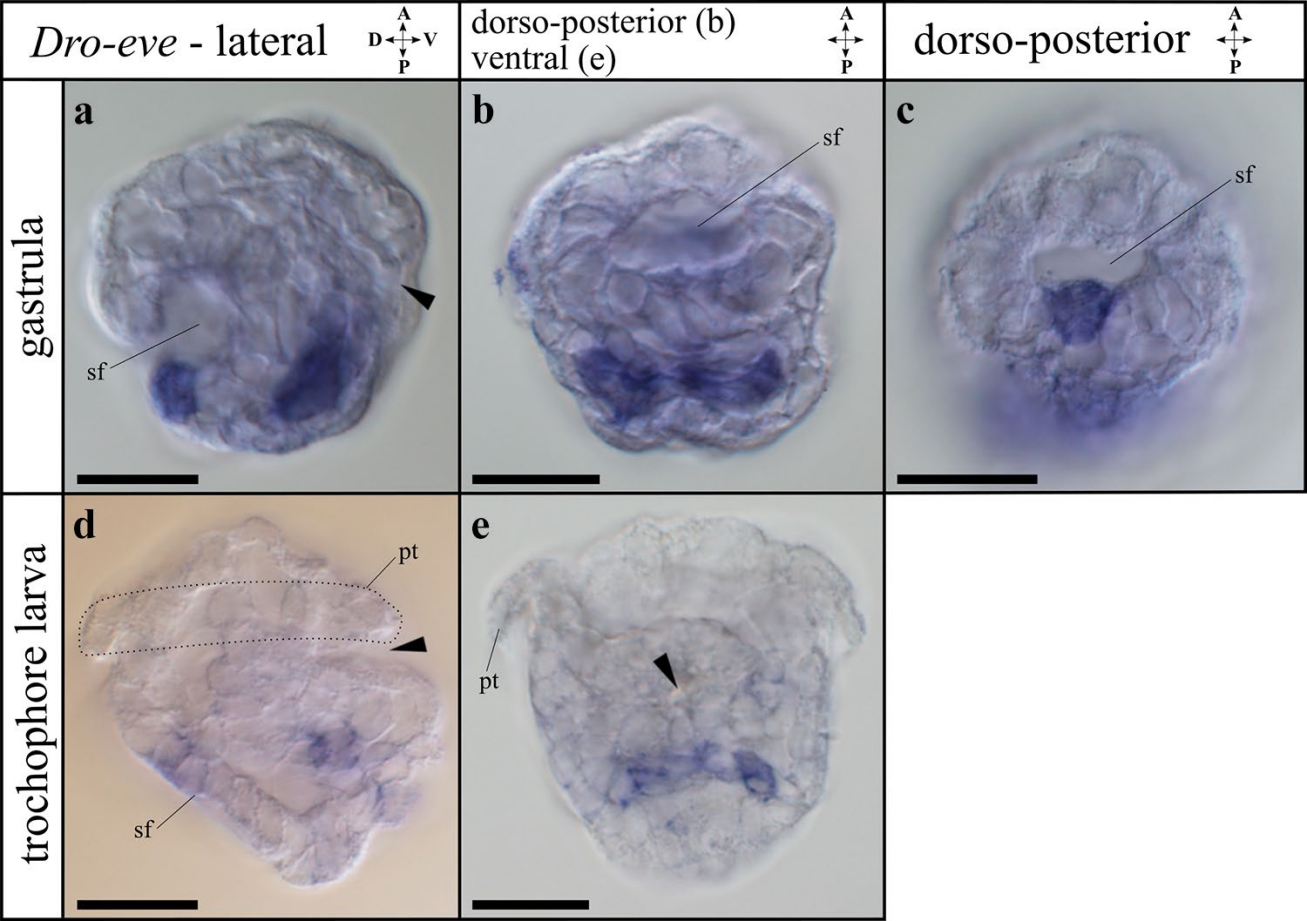
Expression of *even-skipped* (*Dro-eve*) during early development in *Dreissena rostriformis*. Anterior is up. Black arrowheads indicate the blastopore/stomodaeum, dotted line marks the region of the prototroch (pt), and sf marks the shell field. Scale bar equals 20 µm. **a** *Dro-eve* is first expressed in the ventral mesoderm and in ectodermal cells of the shell field. **b** *Dro-eve* expression in the ventral mesoderm ventro-posteriorly of the shell field. **c** Ectodermal expression of *Dro-eve* in the shell field. **d** *Dro-eve* expression in the ventro-posterior mesoderm and in the shell field of the trochophore larva. **e** Mesodermal expression of *Dro-eve* in ventral view. A, anterior; D, dorsal; P, posterior; V, ventral

A single *D. rostriformis Brachyury* (*Dro-Bra*) ortholog nests within the *Brachyury* family, which includes a specific lysine (K) (Conlon et al., 2001; Sebé-Pedrós et al., 2013) at position 121 (Supplemental Fig. 3). Six further candidates (*Eomes*, *Tbx2*, *Tbx3*, *Tbx15*, twice *Tbx20*) with a T-box domain were found in *D. rostriformis* and nest within the corresponding family (Supplemental Fig. 3 and Supplemental Table 7).

**Fig. 3:**
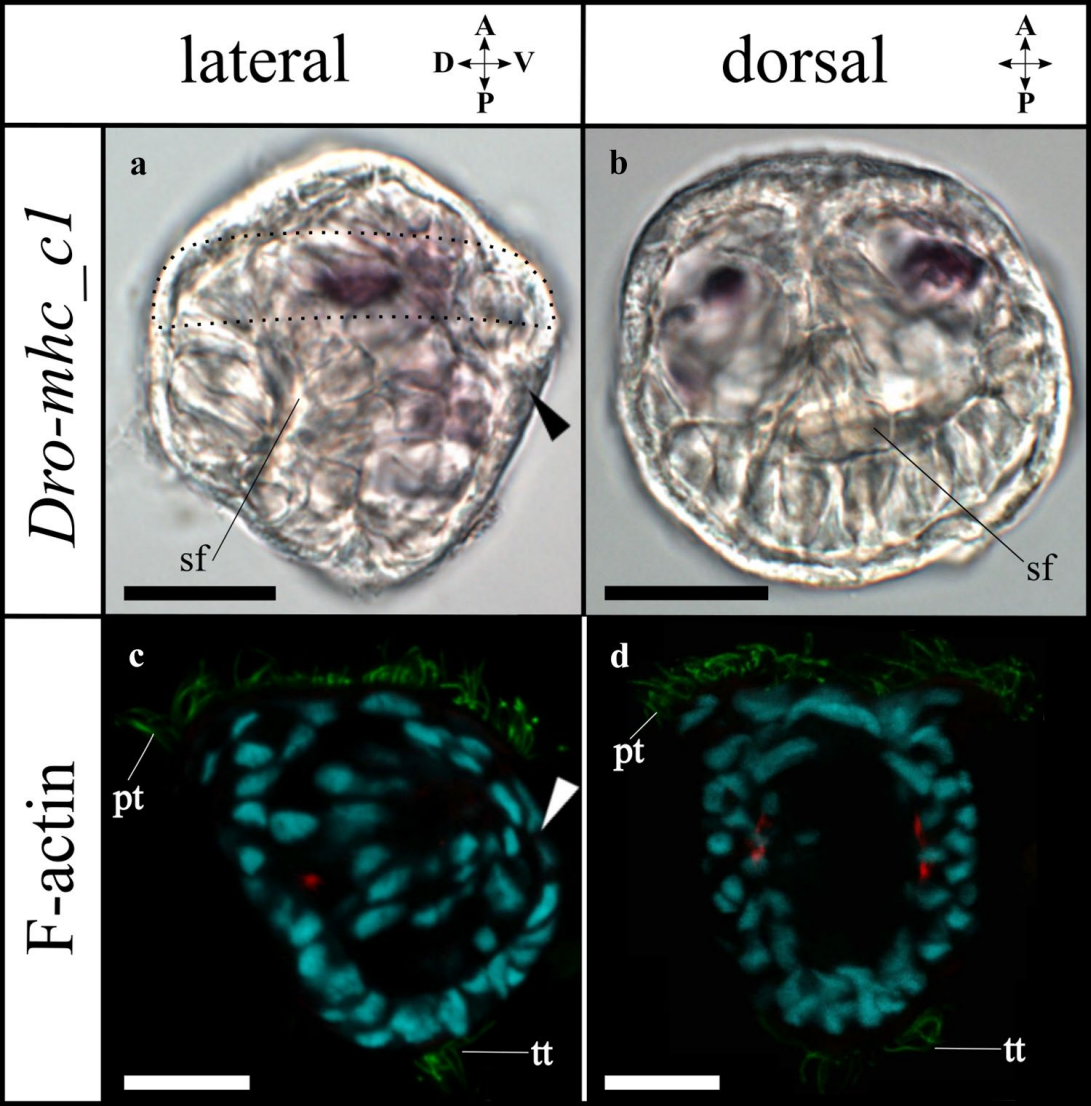
Expression of *myosin II heavy chain* (*Dro-mhc_c1*) and immunofluorescence staining in *Dreissena rostriformis* trochophore larvae. Anterior is up. Arrowheads indicate the stomodaeum, sf marks the shell field, dotted line outlines the region of the prototroch (pt). Scale bar equals 20 µm. Brightfield images (**a**, **b**) of the gene expression and confocal images (**c**, **d**) with F-actin (red), cilia (green; pt: prototroch; tt: telotroch), and cell nuclei staining (cyan). **a** *Dro-mhc_c1* expression is first present in the anterior mesoderm. **b** Anterior mesodermal expression in dorsal view. **c** First F-actin-positive domain in the mesoderm below the shell field in the dorso-median region. **d** Slightly further developed trochophore larva showing two developing myofilaments in the median region. A, anterior; D, dorsal; P, posterior; V, ventral

### Orientation and characteristics of developmental stages

**Fig. 4:**
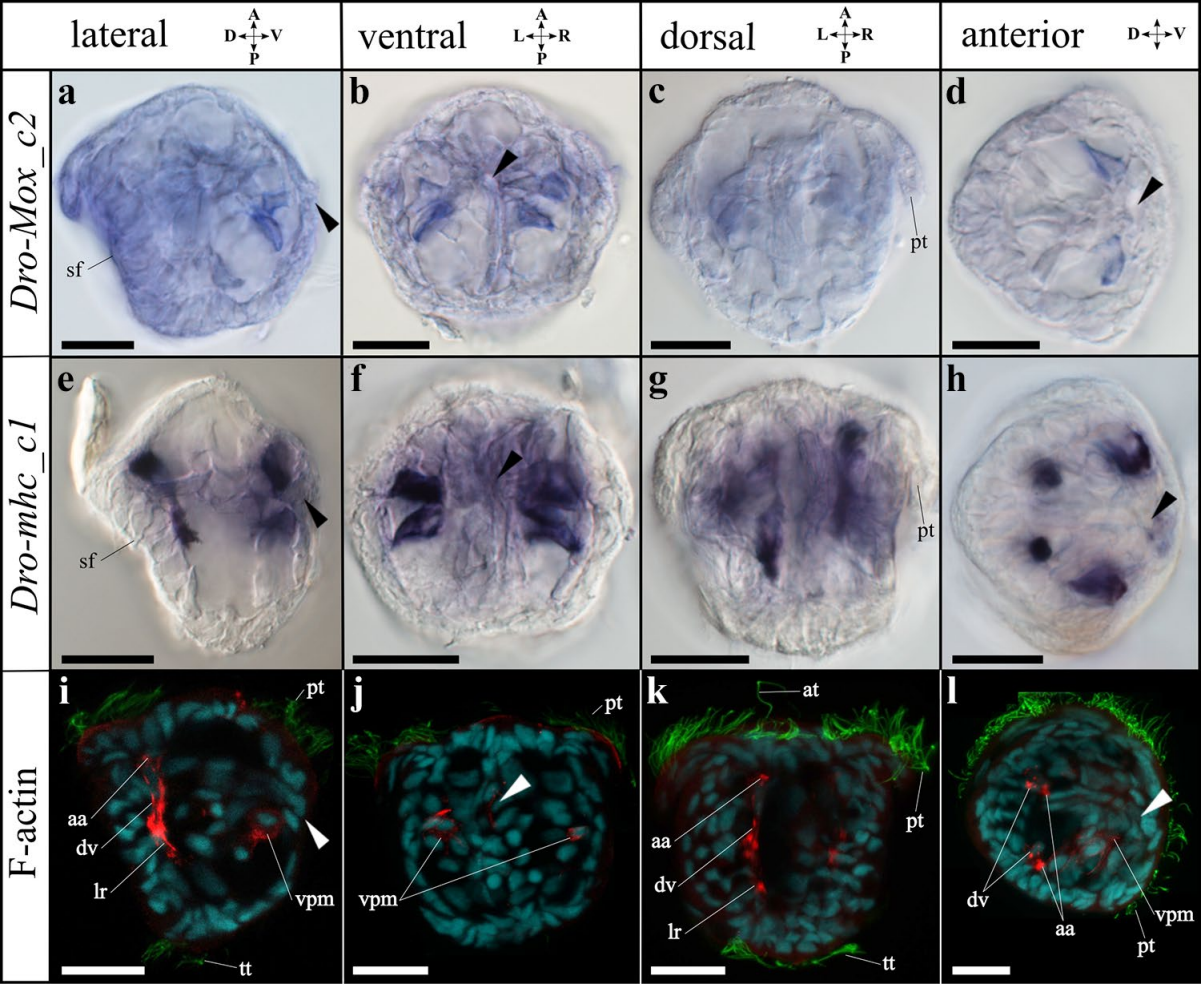
Expression of *Mox* (*Dro-Mox_c2*) and *myosin II heavy chain* (*Dro-mhc_c1*) as well as immunofluorescence staining in *Dreissena rostriformis* trochophore larvae. Anterior is up in all images except **d**, **h**, and **l**, which are anterior views. Arrowheads indicate the stomodaeum, and sf marks the shell field. Scale bar equals 20 µm. Brightfield images (**a**–**h**) of the gene expression and confocal images (**i**–**l**) with F-actin (red), cilia (green; at: apical tuft; pt: prototroch; tt: telotroch), and cell nucleus staining (cyan). **a** Two ventral mesodermal expression domains of *Dro-Mox_c2*. **b** *Dro-Mox_c2* is expressed adjacent to and posterior of the developing digestive tract, on either side. **c** Lack of *Dro-Mox_c2* expression in the dorsal region. **d** Expression of *Dro-Mox_c2* in the ventral mesoderm. **e** *Dro-mhc_c1* is expressed in the dorsal and ventral mesoderm. **f** Four ventral mesodermal expression domains of *Dro-mhc_c1*. **g** Two spot-like and two stripe-like mesodermal expression domains of *Dro-mhc_c1*. **h** Mesodermal expression of *Dro-mhc_c1* in the dorsal and ventral region. **i** Earliest developing myofilaments of the larval retractor (lr), the dorsal velum retractor (dv), the anlage of the anterior adductor (aa), and the ventro-posterior musculature (vpm). **j** The paired ventro-posterior musculature. **k** Developing dorsal velum and larval retractor (dv, lr) and the anlagen of the anterior adductor (aa). **l** Anterior view of developing muscle systems. A, anterior; D, dorsal; L, left; P, posterior; R, right; V, ventral

**Fig. 5:**
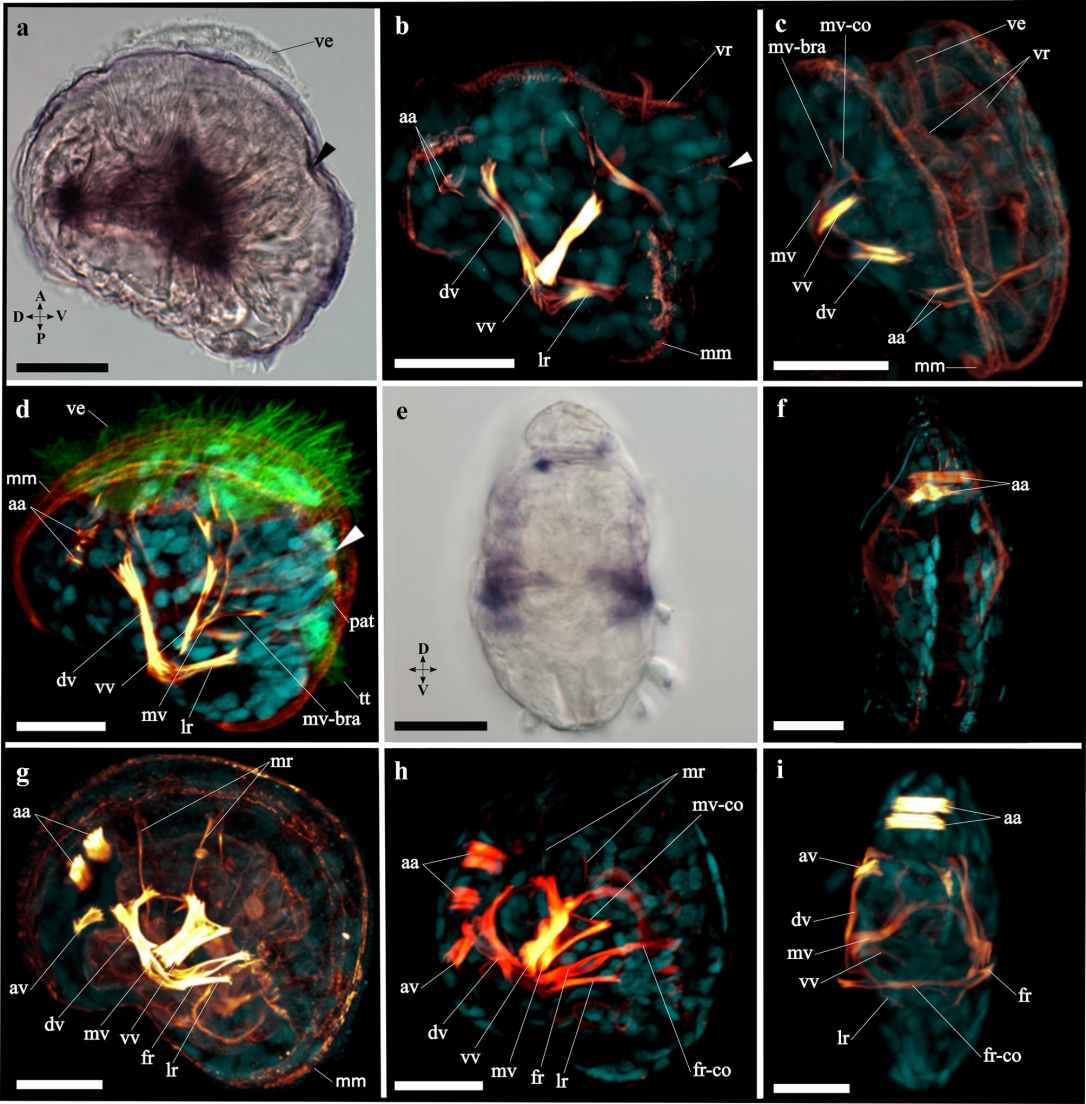
Expression of *myosin II heavy chain* (*Dro-mhc_c1*) and myogenesis in *Dreissena rostriformis* veliger larvae. Lateral view in all images, anterior faces upwards and dorsal to the left except in **c** which is a dorso-anterior view, **e** and **f** which are anterior views (dorsal is up), and **i** which is a posterior view (dorsal is up). Arrowheads indicate the stomodaeum. Scale bar equals 20 µm. Brightfield images of the gene expression (**a** and **e**) and confocal images (**b**–**d** and **f**–**i**) with F-actin (yellow–red), cilia (green), and cell nuclei staining (cyan). **a** Expression of *Dro-mhc_c1* is in the central and dorsal mesoderm. Velum (ve). **b** First distinct muscle bundles are the dorsal velum retractor (dv), the ventral velum retractor (vv), and the larval retractor (lr). First appearance of the velum muscle ring (vr), the (pallial) muscles around the mantle margin (mm), and the merged anterior adductors (aa). **c** D-shaped veliger larva showing the median velum retractor (mv) with a branch (mv-bra) and interconnection (mv-co). **d** Same stage as in **c** with the typical cilia on the velum (ve), pre-anal tuft (pat), and telotroch (tt). **e** *Dro-mhc_c1* in the dorsal and lateral mesoderm of the D-shaped veliger larva. **f** Late veliger larva with prominent anterior adductor (aa). **g** Same stage as in **f** additionally showing the foot retractor (fr), the accessory velum retractor (av), and two mantle retractors (mr). **h** Slightly older late veliger larva as in **g** showing the connection of the foot retractors (fr-co) in the median plane. **i** Posterior view of the same stage as in **h**. A, anterior; D, dorsal; P, posterior; V, ventral

At 18 °C, a free-swimming ciliated gastrula forms by 18 h post fertilisation (hpf). The developing shell field is characterised by a deep invagination on the dorsal side that is surrounded by large ectodermal cells, while the blastopore marks the ventral side (Figs. 1 and 2). By about 24 hpf, the early trochophore larva has developed. Evagination of the shell field commences and is completed by approximately 30 hpf. A ciliated two-rowed prototroch is distinct, together with an apical tuft and a posterior telotroch (Figs. 1, 2, 3, and 4). Between 30 and 40 hpf, an early (D-shaped) veliger larva has developed. It is characterised by two lateral valves that form the embryonic shell (protoconch I: often referred to prodissoconch in bivalves; see Wanninger & Wollesen, 2015) and by a ciliated velum that forms from the prototroch. In addition, *Dreissena* veliger larvae also exhibit a pre-anal tuft on the ventral side, a telotroch on the ventro-posterior side, and a functional digestive tract. After 4–5 days, the D-shape of the veliger larva changes and the umbo begins to form (Fig. 5). The oldest veliger larvae were over 1 month old.

### Developmental expression of *Brachyury* and *even-skipped*

*Dro-Bra* shows high relative expression with respect to other genes during early stages (< 18 hpf), with a considerable relative decrease in the gastrula stage (18–23 hpf) (Supplemental Table 5). Relative expression values remain low in the trochophore (23–30 hpf) and veliger stage (> 30 hpf) (Supplemental Fig. 4a and Supplemental Table 5). *Dro-Bra* expression is first detected in the developing mesoderm in the ventral region of the gastrula (18 hpf) (Fig. 1a, b). In the trochophore larva (30 hpf), *Dro-Bra* is expressed in the endoderm on either side along the invagination of the developing digestive tract (Fig. 1c, d). In addition, expression of *Dro-Bra* is present in the ventro-posterior mesoderm and is located posteriorly to the developing digestive tract in the region of the future hindgut (Fig. 1c, d). No expression of *Dro-Bra* was observed in the veliger larva.

*Dro-eve* transcripts show high relative expression values with respect to other genes in early stages (< 18 hpf), with a considerable decrease from the gastrula stage (18–23 hpf) onwards (Supplemental Fig. 4a and Supplemental Table 5). Using in situ hybridization, *Dro-eve* expression was first detected in the gastrula stage (18 hpf) in three distinct domains. One domain corresponds to the dorsal ectoderm of the shell field (Fig. 2a, c), while the other two are situated ventrally in the developing mesoderm close to the *Dro-Bra* expression domains (Fig. 2a, b). Trochophore larvae (30 hpf) show two *Dro-eve* expression domains, one in the dorsal ectoderm in the median region of the shell field and one in the ventro-posterior mesoderm (Fig. 2d, e). The mesodermal expression of *Dro-eve* is located posteriorly to the developing digestive tract and lies adjacent to the expression of *Dro-Bra*, with the former extending further laterally (Fig. 2d, e). No expression domains of *Dro-eve* were observed in the veliger larva.

### Developmental expression of *myosin II heavy chain*

*Dro-mhc* candidate genes are relatively lowly expressed in early stages (< 18 hpf) with respect to other genes, with a slight relative increase in the gastrula stage (18–23 hpf). This is followed by further relative increases in the trochophore stage (23–30 hpf) and, more prominently, in the veliger stage (> 30 hpf) (Supplemental Fig. 4b and Supplemental Table 5). Two *Dro-mhc_c1* expression domains are first detected in the anterior mesoderm of the early trochophore larva (24 hpf). They are spot-like and situated in the anterior region between the developing digestive tract and the shell field, close to the first F-actin positive cells that appear at ~30 hpf (Fig. 3). In the trochophore larva (30 hpf), four expression domains of *Dro-mhc_c1* are present in the dorsal mesoderm. Two of them are stripe-like and extend along the anterior–posterior axis (Fig. 4e, g). These domains likely give rise to the developing dorsal velum retractors and the larval retractors (Fig. 4i, k). The other two *Dro-mhc_c1* expression domains are spot-like and located in the dorso-anterior region (Fig. 4e, g, h). Their position corresponds to that of the anlagen of the anterior adductors (Fig. 4i, k, l). Additionally, four expression domains are found in the ventral mesoderm, adjacent to and posterior of the developing digestive tract as well as in the region of the ventro-posterior musculature and the *Dro-Mox_c2* domain (Fig. 4). The ventral expression domains of *Dro-mhc_c1* are slightly larger than those of *Dro-Mox_c2* (Fig. 4b, f).

In the D-shaped veliger larva (70 hpf), three expression domains of *Dro-mhc_c1* are present. Two of them are located laterally on both sides of the larva’s median region, at the sites of the velum retractors and larval retractors (Fig. 5). The third expression domain is in the dorsal region between the shell plates, in the region of the developing anterior adductors (Fig. 5).

### *Mox* expression

*Dro-Mox_c2* shows low relative expression levels with respect to other genes and is only briefly upregulated in the trochophore and veliger stages at 26 and 36 hpf, respectively (Supplemental Fig. 4a and Supplemental Table 5). *Dro-Mox_c2* expression is first (and only) detected in the ventral mesoderm of the trochophore larva (30 hpf). Expression of *Dro-Mox_c2* is adjacent to and posterior of the developing digestive tract, on either side (Fig. 4a, b, d). These expression domains correspond to the region of the ventro-posterior musculature and to the ventral expression domain of *Dro-mhc_c1* (Fig. 4). No *Dro-Mox_c2* expression was observed in the veliger larva.

### Myogenesis

**Fig. 6:**
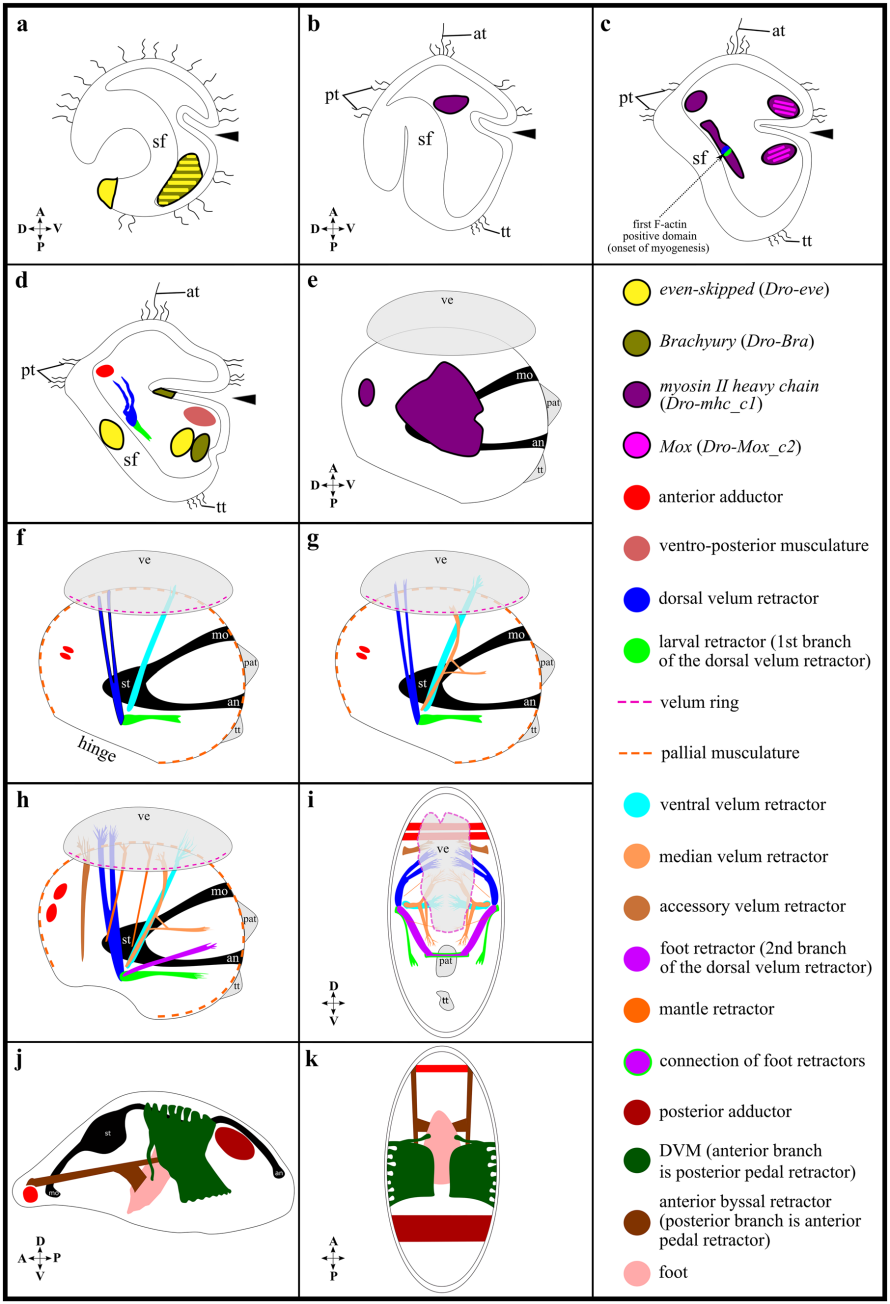
Schematic summary of gene expression and myogenesis in *Dreissena rostriformis*. Lateral view in all images with anterior facing upwards and dorsal to the left except in **i** which is an anterior view and **k** which is a dorsal view. A (anterior), an (anus), apical tuft (at), black arrowheads (blastopore/stomodaeum), D (dorsal), mo (mouth), P (posterior), pre-anal tuft (pat), prototroch (pt), sf (shell field), st (stomach), telotroch (tt), V (ventral), ve (velum). **a** Ciliated gastrula with mesodermal expression of *Brachyury* (*Bra*) and *even-skipped* (*eve*), and ectodermal expression of *eve*. **b** Trochophore larva with mesodermal expression of *myosin II heavy chain* (*mhc*). **c** Late trochophore larva showing mesodermal expression of *Mox* and *mhc*, as well as the first F-actin-positive domain. **d** Trochophore larva with gene expression in the mesoderm (*Bra*, *eve*), endoderm (*Bra*), and ectoderm (*eve*). In addition, the first muscles appear: dorsal velum retractor, larval retractor, anlage of the anterior adductor, and ventro-posterior musculature. **e** D-shaped veliger larva with *mhc* expression in the dorsal and median mesoderm. **f** Same stage as in **e** showing first distinct muscle bundles: dorsal velum retractor, ventral velum retractor, larval retractor, velum muscle ring, pallial musculature, and two-partite anterior adductor. **g** Slightly older stage as in **f** with a median velum retractor. **h** Late veliger larva with additional muscles: accessory velum retractor, foot retractor, and two mantle retractors. **i** Slightly older stage as in **h** showing the connection of both foot retractors. **j, k** Adult *Dreissena* myoanatomy (adapted from Eckroat et al., 1993) including the anterior and the posterior adductor, the dorsal–ventral musculature (DVM), and the anterior byssus retractor

F-actin staining is first detected in the dorso-median mesoderm of the *D. rostriformis* trochophore larva (30 hpf). The paired domains are situated below the median region of the shell field (Figs. 3c, d and 6c). From here, the first pair of myofilaments emerges, which gives rise to the dorsal velum retractors and the developing ventral larval retractors that lie below the shell field. The developing dorsal velum retractors project into the anterior region. In contrast, the developing ventral larval retractors extend into the posterior region with a slightly ventral direction (Figs. 4i, k and 6d). The first anlagen of the anterior adductors also form in the trochophore larva, in the dorso-anterior mesoderm below the shell field, and above the developing dorsal velum retractors (Figs. 4i, k, l and 6d). In addition, a pair of transient ventro-posterior muscles emerges that lies posterior to the developing digestive tract (Figs. 4i, j, l and 6d).

The first distinct muscle bundles develop in the early (D-shaped) veliger larva (40 hpf). The mantle (pallial) musculature is formed around the edges of the mantle (Figs. 5b and 6f). Two fine interconnections of the anterior adductors are visible and attach dorsally to the embryonic shell (Figs. 5b, c and 6f). A pair of ventral larval retractor muscles attaches to the embryonic shell near the hinge and extends ventrally into the region of the hindgut (Figs. 5b and 6f). The velum musculature consists of the newly formed velum muscle ring that underlies the velum (Figs. 5b and 6f). In addition, a pair of dorsal velum retractors inserts posteriorly at the embryonic shell near the hinge and at the dorsal part of the velum. The second pair of velum retractors, the ventral velum retractors, develops between the dorsal velum retractors and the ventral larval retractors and attaches in the median region of the velum and posteriorly at the embryonic shell near the hinge (Figs. 5b, d and 6f). The third pair of velum retractors, the median velum retractors, emerges later in the D-shaped veliger larva. The attachment is posterior to the embryonic shell near the hinge and at the velum between the dorsal velum retractors and the ventral velum retractors (Figs. 5d and 6g). A median branch of the median velum retractors runs towards the ventral part in the region of the hindgut. This branch shows a connection to the median velum retractor (Figs. 5c, h and 6g). In the late veliger larva (102 hpf), the fourth pair of velum retractors (accessory velum retractors), two pairs of mantle retractors, and one pair of foot retractors become visible (Figs. 5g and 6h). The accessory velum retractors are located between the anterior adductors and the dorsal velum retractors. They attach to the most dorsal region of the velum. Two pairs of mantle retractors are situated between the dorsal and the ventral velum retractors, respectively, and both connect to the mantle. The foot retractor emerges as a branch of the dorsal velum retractor and extends into the region of the developing foot (Figs. 5g and 6h). In the late veliger larva, the anterior adductors increase in size. Shortly thereafter, the foot retractors become interconnected and form a U-shape (Figs. 5f, h, i and 6i).

## Discussion

### Comparative *Brachyury* expression in Bilateria

**Fig. 7:**
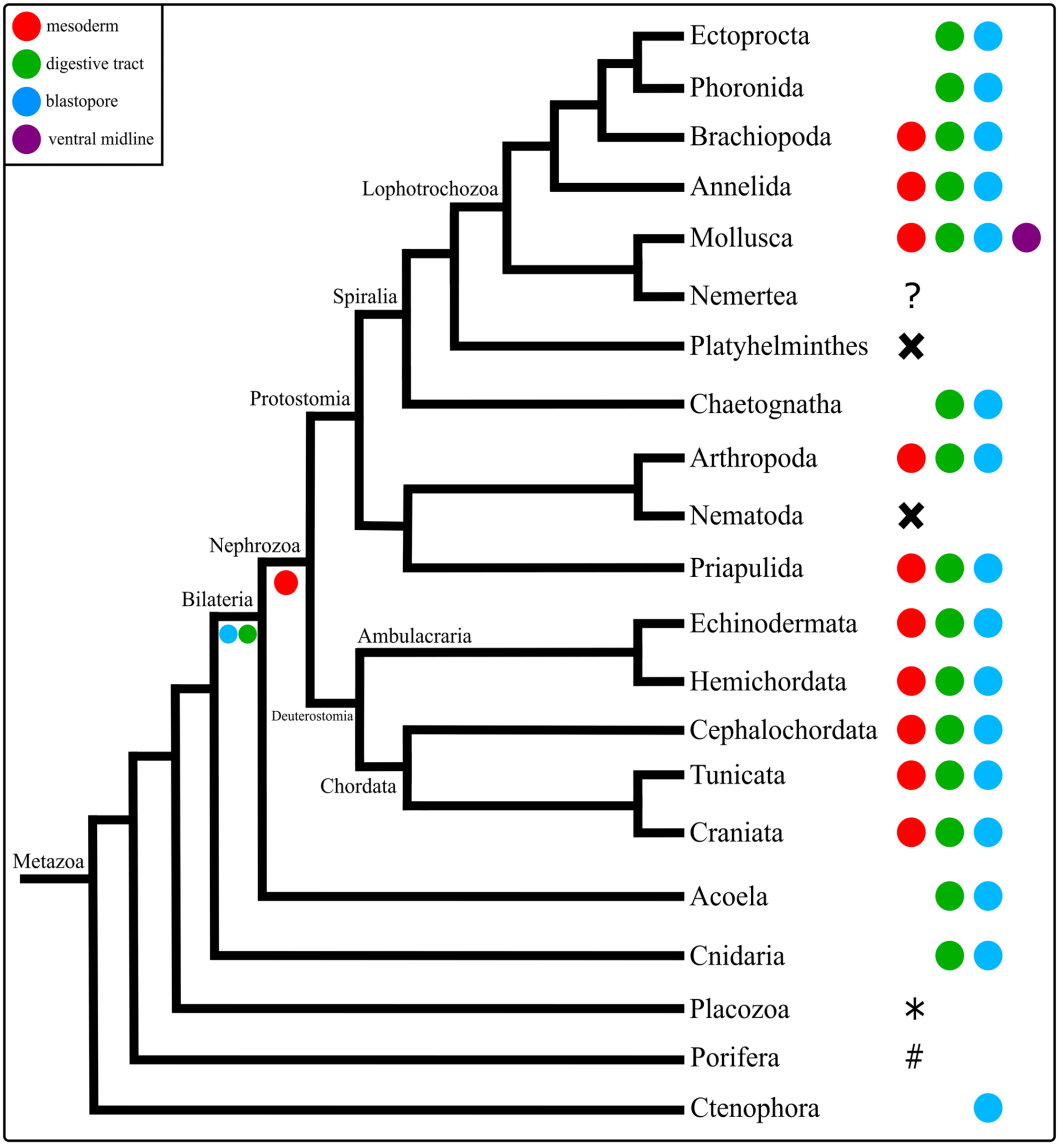
Comparative expression of *Brachyury* (*Bra*) in Metazoa. X: absence of a *Brachyury* ortholog in the genome, ?: no data available, #: *Bra* expression in oocytes, in the choanocytes, and in the choanoderm, *: expression of *Bra* near the edge of potential ‘outgrowth zones’ (Martinelli & Spring, 2003). Phylogeny after Laumer et al. (2019). Comparative analysis implies that *Bra* has a conserved role in digestive tract and blastopore development amongst bilaterian animals and a conserved role in mesoderm formation in nephrozoans. The expression of *Bra* in the ectoderm along the ventral midline is a novelty in mollusks

In *Dreissena rostriformis*, *Bra* is expressed in the developing mesoderm in the gastrula stage and in the trochophore larva, with additional expression in the developing foregut. A very similar *Bra* expression pattern is found in the gastrula of the Pacific oyster *Crassostrea gigas* (Tan et al., 2017). In the spiny oyster *Saccostrea kegaki*, first *Bra* expression is in the vegetal region of the 16-cell stage. After that, *Bra* is also expressed in the putative mesoderm in the ventral region, similar to *C. gigas* and *D. rostriformis*. In *S. kegaki*, *Bra* is additionally expressed in the ectoderm along the ventral midline and near the blastopore. After the evagination of the shell field, *Bra* is restricted to the presumptive anus region, similar to *D. rostriformis*. In contrast to *D. rostriformis*, no *Bra* expression was found in the foregut of *S. kegaki* (Kin et al., 2009). In most gastropods, *Bra* expression is similar to that of bivalves, as it is expressed in the mesoderm and digestive tract, as well as ectodermally near the blastopore and along the ventral midline (Fig. 7; Lartillot et al., 2002; Perry et al., 2015). Since the latter expression domain is only present in mollusks, it seems to be an apomorphy of Mollusca, while the other expression domains also occur in other taxa (Fig. 7).

*Bra* expression has been described near and/or around the blastopore and often also in the digestive tract in a vast number of metazoans (Fig. 7; e.g., Peter & Davidson, 2011; Green & Akam, 2014; Hejnol & Martín-Durán, 2015; Martín-Durán et al., 2017; Sebé-Pedrós & Ruiz-Trillo, 2017). This indicates that *Bra* appears to have a conserved role in blastopore and digestive tract formation amongst bilaterian animals (Fig. 7). Mesodermal expression of *Bra* has been reported in most nephrozoan taxa (protostomes and deuterostomes), except for a few spiralians, e.g., ectoprocts, phoronids, and chaetognaths, where *Bra* expression was not detected in the mesoderm (Fig. 7; Andrikou et al., 2019; Green & Akam, 2014; Hejnol & Martín-Durán, 2015; Kusch & Reuter, 1999; Martín-Durán et al., 2012, 2017; Nishino et al., 2001; Perry et al., 2015; Peter & Davidson, 2011; Peterson et al., 1999; Satou & Imai, 2015; Takada et al., 2002; Terazawa & Satoh, 1997; Vellutini et al., 2017). Since mesodermal expression of *Bra* appears to be absent in the acoel *Convolutriloba longifissura*, expression of *Bra* in the mesoderm may have evolved in the lineage leading to the nephrozoans, with a possible loss of function in various spiralians and nematodes, whereby *Bra* is absent from the genome of *Caenorhabditis elegans* altogether (Fig. 7; Hejnol & Martindale, 2008a; Martín-Durán & Romero, 2011; Pocock et al., 2004; Sebé-Pedrós & Ruiz-Trillo, 2017). Accordingly, the data currently available suggest that *Brachyury* was expressed during blastopore and digestive tract development in the last common ancestor (LCA) of Bilateria. In addition, *Brachyury* was likely involved in mesoderm formation in the LCA of Nephrozoa, with a novel expression of *Bra* along the ventral midline in the molluscan ectoderm (Fig. 7).

### Comparative *even-skipped* expression in Metazoa

**Fig. 8:**
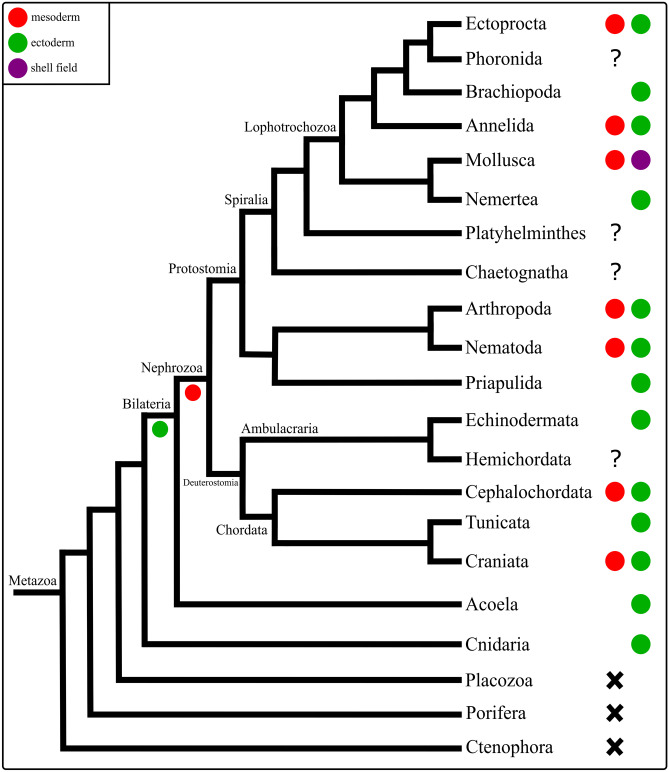
Comparative expression of *even-skipped* (*eve*) in Metazoa. X: absence of an *eve* ortholog in the genome, ?: no data available. Phylogeny after Laumer et al. (2019). The data currently available indicate that *eve* was expressed during ectoderm development in the last common ancestor (LCA) of Bilateria, while *eve* was additionally involved in mesoderm formation in the LCA of Nephrozoa. Expression of *eve* in the ectodermal shell field is an evolutionary novelty of Mollusca

In the gastrula and the trochophore larva of *Dreissena rostriformis*, *eve* is found in the developing mesoderm and in the ectoderm of the shell field. These constitute the first *eve* expression data for any mollusk. In a number of bilaterians and the cnidarian *Nematostella vectensis*, *eve* is expressed in the ectoderm, which is commonly associated with hindgut formation and neurogenesis (Fig. 8; Ikuta et al., 2004; Martín-Durán et al., 2017; Ryan et al., 2007; Vellutini et al., 2017). Accordingly, ectodermal expression of *eve* seems to be a conserved feature across pan-bilaterian taxa (Fig. 8).

Expression of *eve* during mesoderm formation has been documented in vertebrates and the cephalochordate amphioxus, as well as in the majority of protostomes, including most annelids, an ectoproct, *C*. *elegans*, and arthropods (Fig. 8; Ruiz et al., 1989; Ferrier et al., 2001; Seebald & Szeto, 2011; Kozin et al., 2016; Martín-Durán et al., 2017; Vellutini et al., 2017). In *Artemia franciscana*, *Drosophila*, and *C*. *elegans*, *eve* is additionally expressed in mesoderm derivatives such as muscle and/or heart cells, and *eve* expression is required for limb development in the mouse and zebrafish (Ahringer, 1996; Copf et al., 2003; Fujioka et al., 2005; Hérault et al., 1996; Sordino et al., 1996). However, *eve* was not found to be expressed in the mesoderm in a few protostomes, including brachiopods, a nemertean, and a priapulid, as well as in some deuterostomes, e.g., a sea urchin and the ascidian *Ciona intestinalis* (Fig. 8; Ikuta et al., 2004; Li et al., 2014; Martín-Durán & Hejnol, 2015; Martín-Durán et al., 2015, 2017). Since *eve* is neither expressed in the mesoderm of the acoel *C*. *longifissura*, it appears that *eve* may have evolved a role in mesoderm formation only after the acoel-nephrozoan split with loss of function in multiple lineages. This notion is further supported by absence of the *even-skipped* gene in ctenophores, placozoans, and poriferans (Fig. 8; Hejnol & Martindale, 2008b; Leininger et al., 2014; Ryan et al., 2010; Schierwater et al., 2008).

### Comparative *Mox* expression in Metazoa

**Fig. 9:**
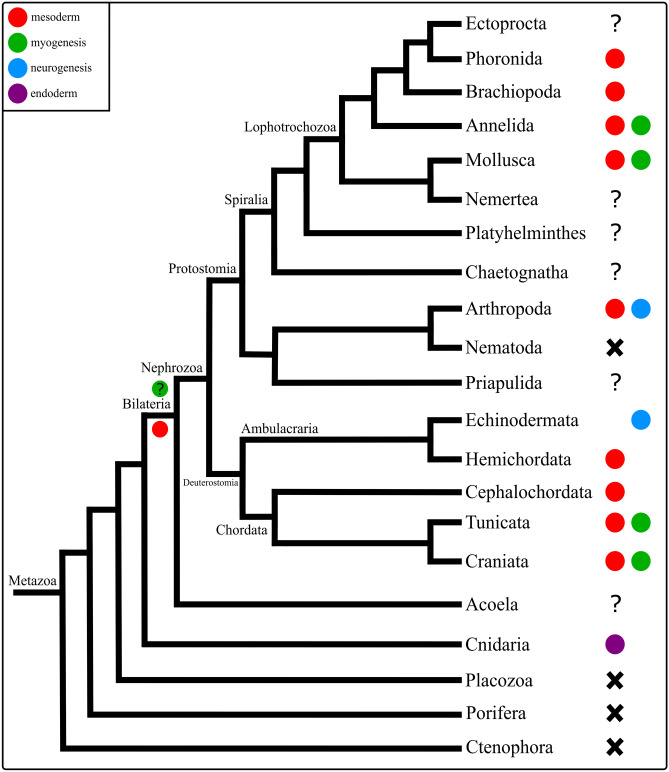
Comparative expression of *Mox* in Metazoa. X: absence of a *Mox* ortholog in the genome, ?: no data available. Phylogeny after Laumer et al. (2019). Comparative analysis suggests that *Mox* has an ancestral role in mesoderm and possibly muscle formation in Bilateria. Expression of *Mox* during neurogenesis has evolved independently in arthropods and echinoderms

In the trochophore larva of *Dreissena rostriformis*, *Mox* is expressed in the ventral mesoderm in the region of the ventral *mhc* domain and at the site of the developing ventro-posterior musculature. Mesodermal and/or muscular *Mox* expression is also found in other mollusks, lophotrochozoans, protostomes, chordates, and cnidarians, suggesting that *Mox* might have already played a role in their development in the LCA of nephrozoans and cnidarians (Fig. 9; Andrikou & Hejnol, 2021; Candia & Wright, 1995; Chiang et al., 1994; Chiori et al., 2009; Hinman & Degnan, 2002; Ikuta et al., 2004; Kozin et al., 2016; Lowe et al., 2006; Mankoo et al., 1999; Minguillón & Garcia-Fernàndez, 2002; Neyt et al., 2000; Passamaneck et al., 2015; Rallis et al., 2001; Ryan et al., 2007; Satou & Imai, 2015). The apparent lack of *Mox* in ctenophores, placozoans, poriferans, and *C. elegans* suggests that this gene family emerged at the base of the eumetazoan lineage with secondary loss in the nematode (Fig. 9; Ruvkun & Hobert, 1998; Ryan et al., 2010; Schierwater et al., 2008).

In the sea urchin embryo and in *Drosophila*, *Mox* is involved in neurogenesis (Fig. 9; Chiang et al., 1994; Poustka et al., 2007). It thus appears likely that *Mox* expression in neural cells may have evolved independently in these lineages, but the database is as of yet too scarce to unequivocally resolve this issue.

### Comparative larval myoanatomy in Bivalvia

**Fig. 10:**
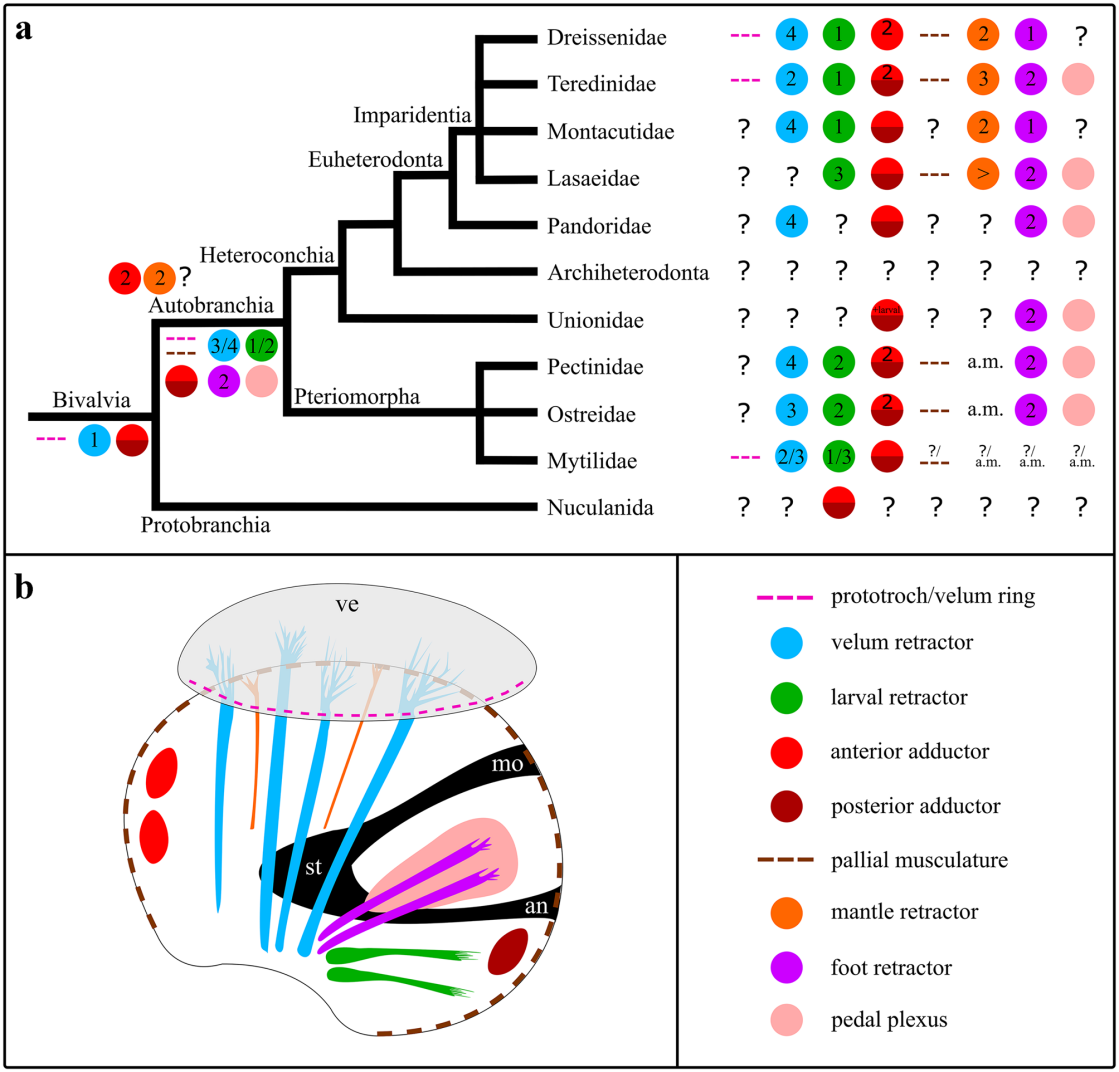
Muscle systems in bivalve lineages. **a** Bivalve phylogeny (after Combosch et al. (2017)) with larval muscle systems in various clades. ?: unknown, numbers: number of paired retractors/adductors, > : set of paired mantle retractors, a.m.: after metamorphosis. Colour code indicates individual muscle systems. Comparative analysis implies that five major muscle systems were present in the last common ancestor (LCA) of autobranch bivalve larvae: The velum musculature including three or four pairs of velum retractors and a velum muscle ring, the larval retractors (one or two pairs), the adductor system containing the anterior as well as the posterior adductor, the mantle musculature including the muscles of the pallial line and possibly two pairs of mantle retractors, and the foot musculature containing two pairs of foot retractors together with the pedal plexus. The data presently available suggest that the muscular ground pattern of bivalve larvae includes at least one pair of velum retractors, a velum muscle ring, and the anterior and the posterior adductor. **b** Schematic drawing of the hypothetical larval myoanatomy in the LCA of autobranch bivalves. Note that the exact number of velum, larval, and mantle retractors remains unclear for the LCA of autobranch bivalve larvae. an, anus; mo, mouth; st, stomach; ve, velum

Five distinct muscle systems are present in the veliger larva of *Dreissena rostriformis*, namely the velum muscle ring, four pairs of velum retractors, one pair of ventral larval retractor, one pair of foot retractor, the mantle musculature including the muscles of the pallial line, and two pairs of mantle retractors, as well as an initially paired anterior adductor (Figs. 6 and 10). A posterior adductor muscle and pedal plexus (foot musculature), as present in the adult, were not found, which most likely emerge in late larval stages or after metamorphosis.

The velum muscle ring degenerates prior to or at metamorphosis and has been reported in dreissenids, teredinids, and mytilids but not in other bivalve larvae (Fig. 10a; Audino et al., 2015; Dyachuk & Odintsova, 2009; Kurita et al., 2016; Li et al., 2019; Sun et al., 2020; Wurzinger-Mayer et al., 2014). However, since the prototroch/velum muscle ring occurs in almost all class-level sublineages of mollusks with indirect development except for the scaphopods, it seems most likely that it is part of the molluscan—and thus also the bivalve—larval muscular ground pattern (Fig. 10a; Wanninger & Wollesen, 2015).

The velum retractors have been documented in all veliger larvae of autobranch bivalves investigated to date and are resorbed prior to or during metamorphosis. Their number differs between species; e.g., four pairs are common in euheterodonts, except for the teredinid shipworm *Lyrodus pedicellatus*, where two pairs are present (Fig. 10a; Wurzinger-Mayer et al., 2014). Interestingly, the two velum retractor pairs of the shipworm were suggested to transform into the future mantle musculature. However, this condition has not been described for any other mollusk and, if true, most likely constitutes an apomorphy of this genus or species (Wurzinger-Mayer et al., 2014). In pteriomorph larvae, four pairs of velum retractors were found in pectinids, whereas three pairs are present in oysters and two to three pairs were described in mytilids (Fig. 10a; Audino et al., 2015; Cragg, 1985; Dyachuk & Odintsova, 2009; Kurita et al., 2016; Li et al., 2019; Sun et al., 2019, 2020). Accordingly, three or four pairs of velum retractors appear most likely to be a part of the myoanatomical ground pattern in autobranch bivalve larvae (Fig. 10).

The larval retractors disappear prior to or at metamorphosis and are present in most autobranch bivalve lineages, even in the semi-direct (brooding) lasaeids (Altnöder & Haszprunar, 2008). However, the number of larval retractors differs amongst species; e.g., one (ventral) pair is common in imparidents, except in the lasaeids which contain three pairs, while in pteriomorphs, one to five pairs are present (Fig. 10a; Audino et al., 2015; Kurita et al., 2016; Li et al., 2019; Sun et al., 2020; Wurzinger-Mayer et al., 2014). Accordingly, one or two pairs of larval retractors appear most likely to be a part of the muscular ground pattern in autobranch bivalve larvae (Fig. 10).

A dimyarian condition, i.e. the presence of an anterior and a posterior adductor muscle, is common for many adult bivalves. They are usually formed in the larva and are transiently present in pectinid and oyster larvae that as adults only have one adductor. Here, the adult monomyarian condition is achieved by loss of the anterior adductor at metamorphosis (Fig. 10a; Audino et al., 2015; Cragg, 2016; Drew, 1899, 1901; Li et al., 2019; Sun et al., 2019, 2020; Wurzinger-Mayer et al., 2014). Interestingly, a transient larval adductor is also present in the parasitic glochidium larva of unionids, but the adult anterior and posterior adductors appear to develop independently during metamorphosis (Herbers, 1913).

In most bivalves, the muscles of the pallial line develop in later larval stages while the paired (adult) mantle retractors are formed after metamorphosis. Their number differs amongst species; e.g., two pairs of retractors are present in dreissenids and montacutids, while three pairs were found in the teredinids (Fig. 10a; Dyachuk & Odintsova, 2009; Li et al., 2019; Sun et al., 2020; Wurzinger-Mayer et al., 2014). This variation is also found in the (adult) foot retractors, where one pair is present in dreissenids and montacutids, while two pairs are common in most other autobranchs (Fig. 10a).

Taken together, it appears that at least a velum muscle ring, three or four pairs of velum retractors, one or two pairs of larval retractors, an anterior and a posterior adductor, and two pairs of foot retractors together with the plexus-like foot musculature as well as the mantle musculature including muscles of the pallial line and possibly two pairs of mantle retractors, are part of the muscular ground pattern of autobranch bivalve larvae (Fig. 10). The two-partite condition of the anterior adductor in early development throughout Autobranchia might argue for a paired anterior adductor in the LCA of autobranchs or even Bivalvia. For further assessments concerning the ground plan of the entire Bivalvia, more data on the Protobranchia, the sister taxon to all other bivalves, are required.

## Conclusion

The present study shows that expression of *Bra*, *eve*, and *Mox* in the quagga mussel *Dreissena rostriformis* is congruent with numerous other bilaterian taxa. The data currently available suggest that *Mox* had an ancestral role in bilaterian mesoderm formation, while *even-skipped* and *Brachyury* have obtained their mesodermal expression domains after the xenacoelomorph-nephrozoan split. The data on bivalve myogenesis indicate that the muscular ground pattern of autobranch—and maybe even all—bivalve larvae contains a highly complex arrangement of larval retractor muscles and heterochronically shifted, functional adult systems that undergo significant, taxon-specific remodelling and reduction events during metamorphosis.

## Supplementary Information

Below is the link to the electronic supplementary material.

Supplementary file1 (TIFF 1095 kb)

Supplementary file2 (TIFF 1079 kb)

Supplementary file3 (TIFF 949 kb)

Supplementary file4 (TIFF 855 kb)

Supplementary file5 (DOCX 16 kb)

Supplementary file6 (DOCX 15 kb)

Supplementary file7 (DOCX 16 kb)

Supplementary file8 (DOCX 13 kb)

Supplementary file9 (DOCX 15 kb)

Supplementary file10 (DOCX 14 kb)

Supplementary file11 (DOCX 14 kb)

## Data Availability

All key data generated and/or analysed during the current study are included in this manuscript. Additional data are available from the corresponding author on reasonable request.
