# Supplementary figures and images for: A mosaic of conserved and novel modes of gene expression and morphogenesis in mesoderm and muscle formation of a larval bivalve
Source: Org Divers Evol. 2022 Jul 7;22(4):893–913. doi: 10.1007/s13127-022-00569-5 (PMC9649484; doi:10.1007/s13127-022-00569-5)

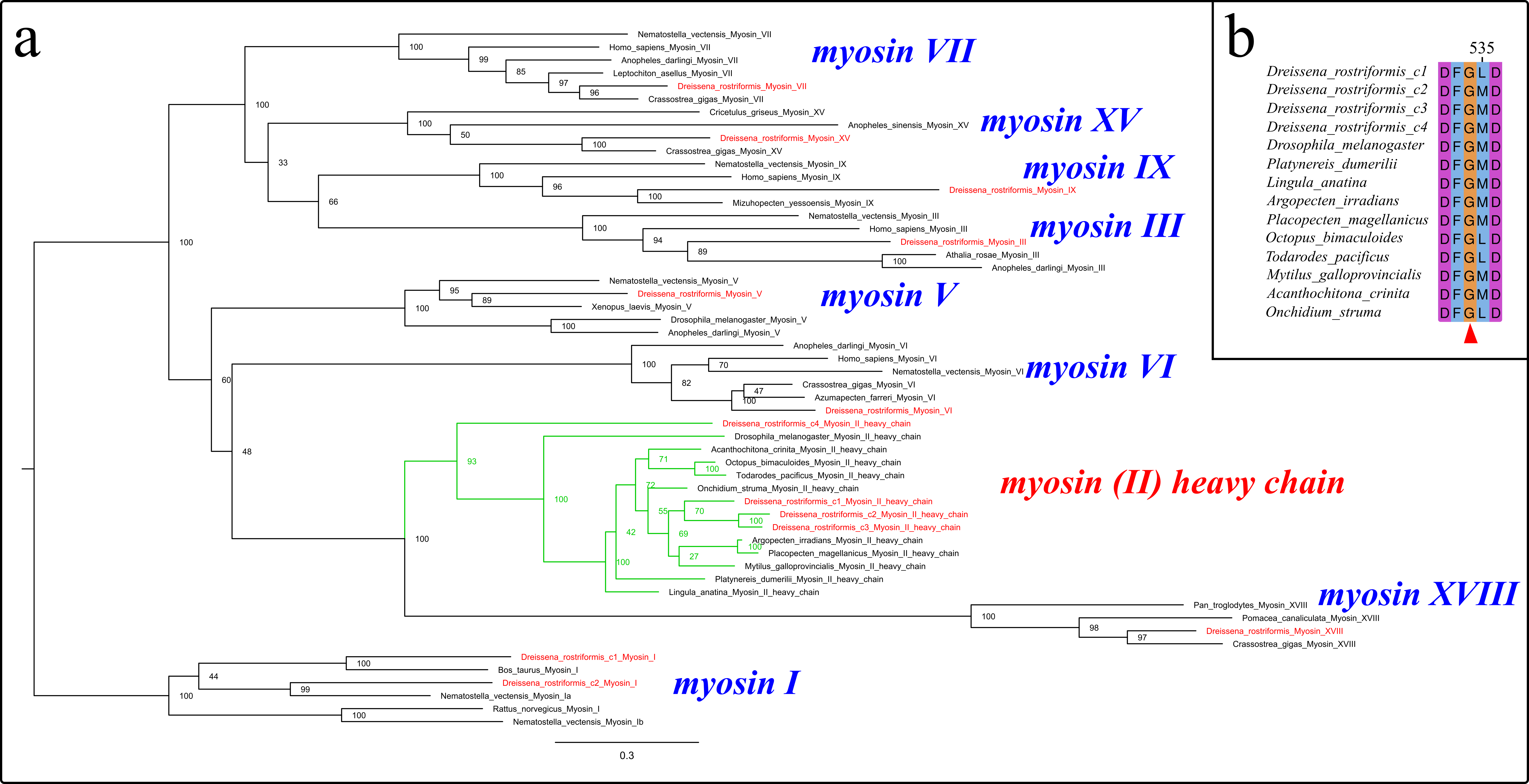

Supplement: Supplementary file 1 — Supplementary file1 (TIFF 1095 kb) [file 13127_2022_569_MOESM1_ESM.tiff]

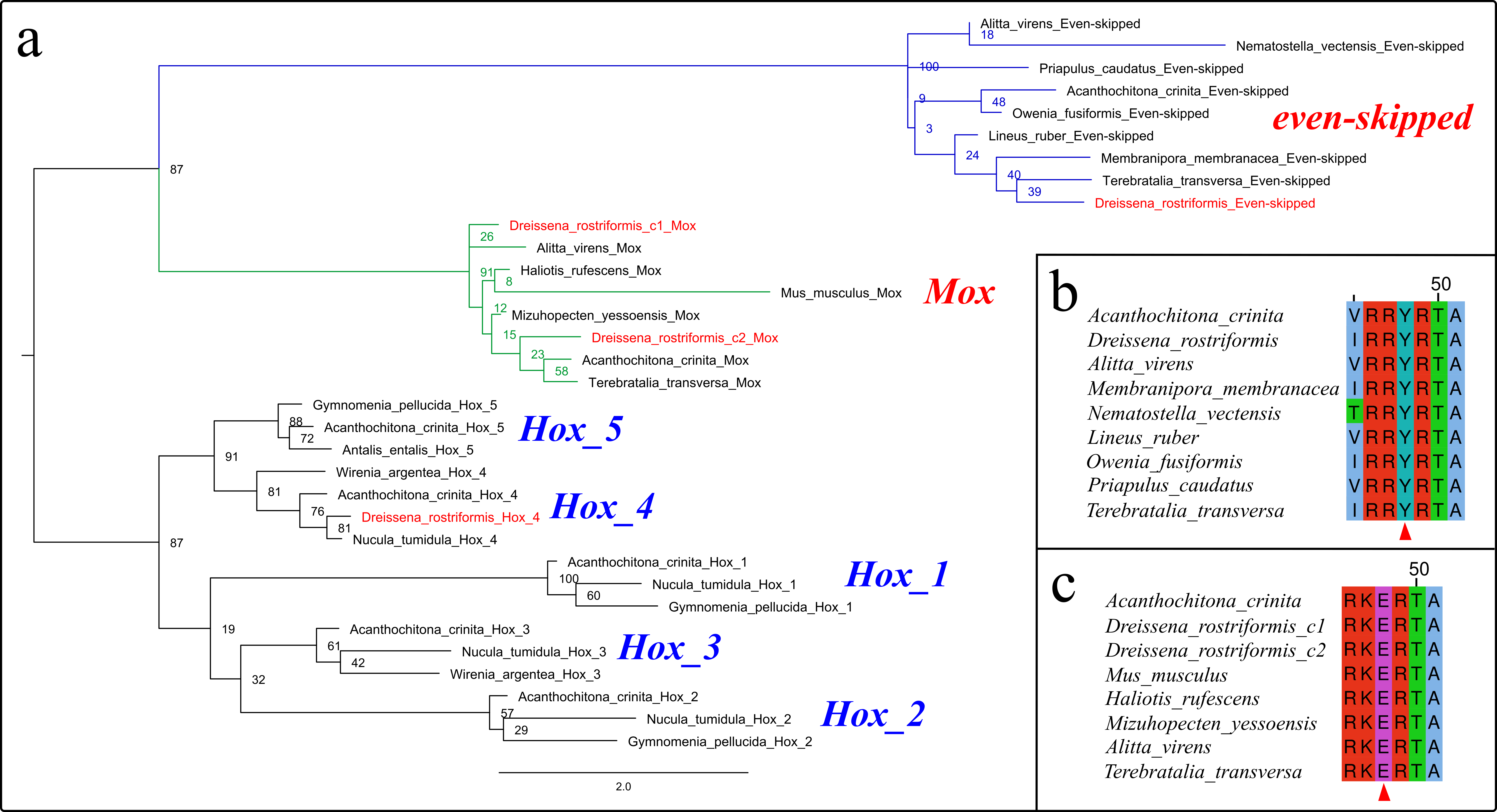

Supplement: Supplementary file 2 — Supplementary file2 (TIFF 1079 kb) [file 13127_2022_569_MOESM2_ESM.tiff]

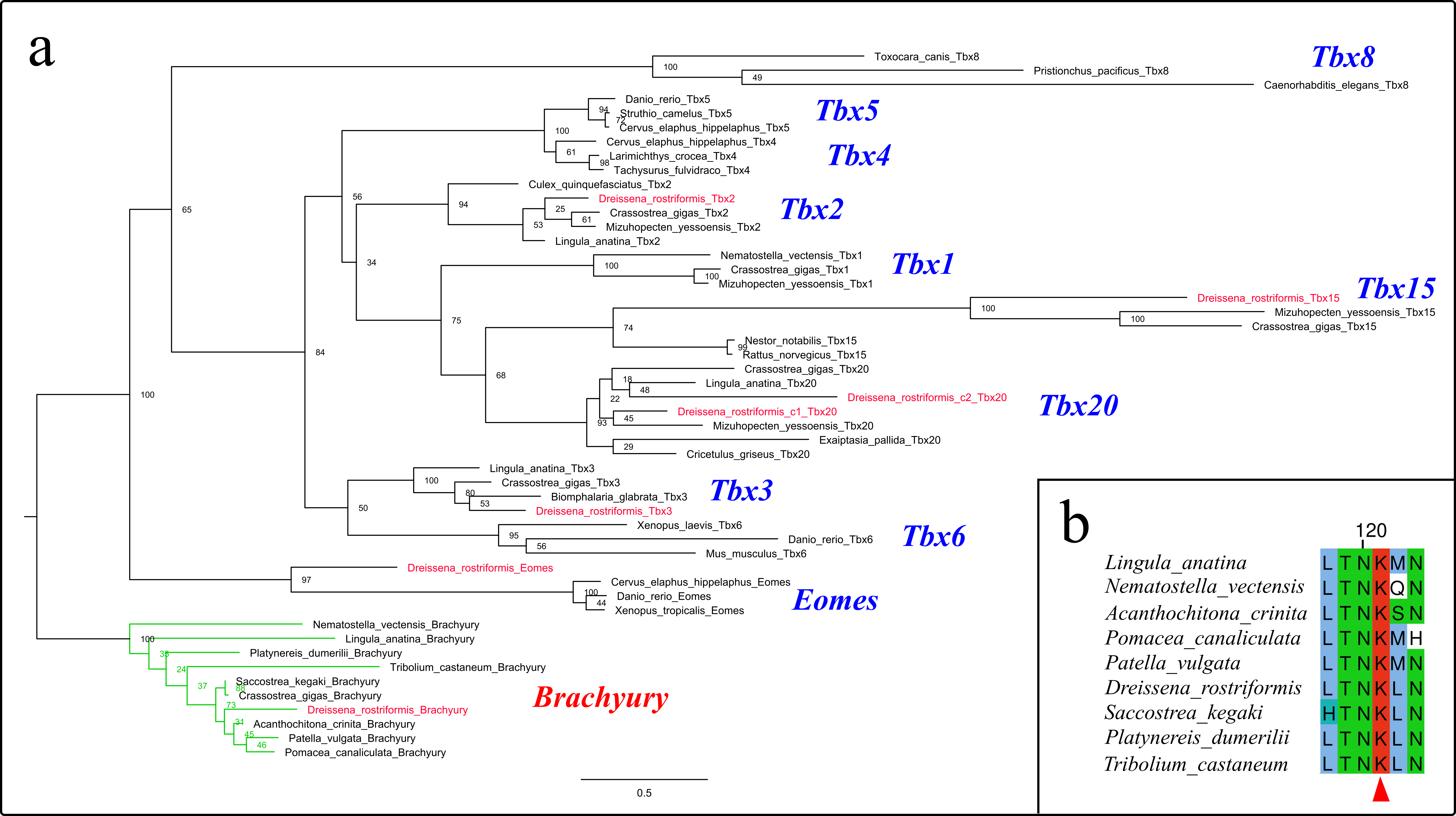

Supplement: Supplementary file 3 — Supplementary file3 (TIFF 949 kb) [file 13127_2022_569_MOESM3_ESM.tiff]

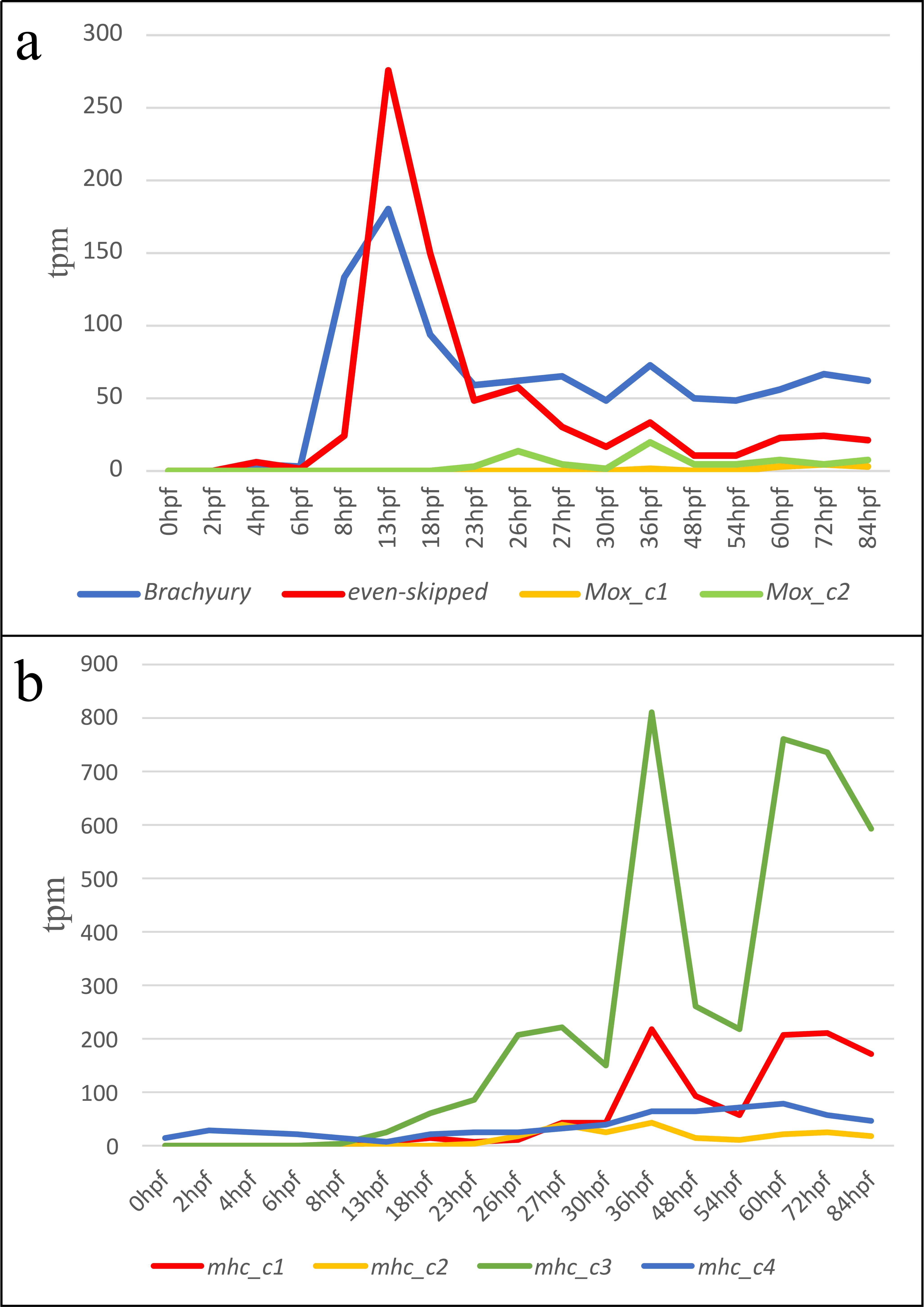

Supplement: Supplementary file 4 — Supplementary file4 (TIFF 855 kb) [file 13127_2022_569_MOESM4_ESM.tiff]
