# Supplementary material for: A mosaic of conserved and novel modes of gene expression and morphogenesis in mesoderm and muscle formation of a larval bivalve
Source: Org Divers Evol. 2022 Jul 7;22(4):893–913. doi: 10.1007/s13127-022-00569-5 (PMC9649484; doi:10.1007/s13127-022-00569-5)
Supplement: Supplementary file 5 — Supplementary file5 (DOCX 16 kb) [file 13127_2022_569_MOESM5_ESM.docx]

| **Gene** | **species name (same in tree)** | **NCBI accession number / Ensembl Metazoa accession number *** |
| --- | --- | --- |
| *myosin II heavy chain (mhc)* | *Dreissena rostriformis c1* | GHRL01011381 |
| *Mhc* | *Dreissena rostriformis c2* | GHRL01034471 |
| *Mhc* | *Dreissena rostriformis c3* | GHRL01017649 |
| *Mhc* | *Dreissena rostriformis c4* | GHRL01006544 |
| *Mhc* | *Acanthochitona crinita (fascicularis)* | GJJB01007089.1 |
| *Mhc* | *Mytilus galloprovincialis* | CAB64662.1 |
| *Mhc* | *Argopecten irradians* | AAC46490.1 |
| *Mhc* | *Placopecten magellanicus* | AAB03661.1 |
| *Mhc* | *Octopus bimaculoides* | CDG41623.1 |
| *Mhc* | *Todarodes pacificus* | ADU19853.1 |
| *Mhc* | *Onchidium struma* | AOR06339.1 |
| *Mhc* | *Lingula anatine* | XP_023932278.1 |
| *Mhc* | *Platynereis dumerilii* | AIJ28480.1 |
| *Mhc* | *Drosophila melanogaster* | NP_523587.4 |
| *myosin I* | *Dreissena rostriformis c1* | GHRL01034922 |
| *myosin I* | *Dreissena rostriformis c2* | GHRL01003178 |
| *myosin I* | *Nematostella vectensis Ia* | * EDO49515 |
| *myosin I* | *Nematostella vectensis Ib* | * EDO45835 |
| *myosin I* | *Rattus norvegicus* | CAA50871.1 |
| *myosin I* | *Bos taurus* | AAA17565.1 |
| *myosin III* | *Dreissena rostriformis* | GHRL01023082 |
| *myosin III* | *Nematostella vectensis* | * EDO43631 |
| *myosin III* | *Anopheles darlingi* | ETN67236.1 |
| *myosin III* | *Athalia rosae* | XP_012256574.1 |
| *myosin III* | *Homo sapiens* | NP_059129.3 |
| *myosin V* | *Dreissena rostriformis* | GHRL01001522 |
| *myosin V* | *Nematostella vectensis* | * EDO41819 |
| *myosin V* | *Xenopus laevis* | AFU81219.2 |
| *myosin V* | *Drosophila melanogaster* | AAC99496.1 |
| *myosin V* | *Anopheles darlingi* | ETN63934.1 |
| *myosin VI* | *Dreissena rostriformis* | GHRL01023773 |
| *myosin VI* | *Nematostella vectensis* | * EDO36037 |
| *myosin VI* | *Azumapecten farreri* | AAY63881.1 |
| *myosin VI* | *Crassostrea gigas* | * EKC25309.1 |
| *myosin VI* | *Anopheles darlingi* | ETN65262.1 |
| *myosin VI* | *Homo sapiens* | AAK00229.1 |
| *myosin VII* | *Dreissena rostriformis* | GHRL01032474 |
| *myosin VII* | *Nematostella vectensis* | * EDO45563 |
| *myosin VII* | *Crassostrea gigas* | * EKC28928.1 |
| *myosin VII* | *Leptochiton asellus* | ASM47588.1 |
| *myosin VII* | *Anopheles darlingi* | ETN66322.1 |
| *myosin VII* | *Homo sapiens* | AAB03679.1 |
| *myosin IX* | *Dreissena rostriformis* | GHRL01016224 |
| *myosin IX* | *Nematostella vectensis* | * EDO38834.1 |
| *myosin IX* | *Mizuhopecten yessoensis* | XP_021359286.1 |
| *myosin IX* | *Homo sapiens* | AAI40870.1 |
| *myosin XV* | *Dreissena rostriformis* | GHRL01005003 |
| *myosin XV* | *Crassostrea gigas* | * EKC41639.1 |
| *myosin XV* | *Cricetulus griseus* | EGW01271.1 |
| *myosin XV* | *Anopheles sinensis* | KFB48001.1 |
| *myosin XVIII* | *Dreissena rostriformis* | GHRL01021375 |
| *myosin XVIII* | *Crassostrea gigas* | * EKC35139.1 |
| *myosin XVIII* | *Pomacea canaliculate* | XP_025090257.1 |
| *myosin XVIII* | *Pan troglodytes* | JAA31795.1 |
