# Supplementary material for: A mosaic of conserved and novel modes of gene expression and morphogenesis in mesoderm and muscle formation of a larval bivalve
Source: Org Divers Evol. 2022 Jul 7;22(4):893–913. doi: 10.1007/s13127-022-00569-5 (PMC9649484; doi:10.1007/s13127-022-00569-5)
Supplement: Supplementary file 6 — Supplementary file6 (DOCX 15 kb) [file 13127_2022_569_MOESM6_ESM.docx]

| **Gene** | **species name (same in tree)** | **NCBI accession number** |
| --- | --- | --- |
| *even-skipped* | *Dreissena rostriformis* | GHRL01002417 |
| *even-skipped* | *Acanthochitona crinita (fascicularis)* | GJJB01012113.1 |
| *even-skipped* | *Alitta virens* | AOS87315.1 |
| *even-skipped* | *Membranipora membranacea* | ARJ36942.1 |
| *even-skipped* | *Nematostella vectensis* | SJX71987.1 |
| *even-skipped* | *Lineus ruber* | AMR72025.1 |
| *even-skipped* | *Owenia fusiformis* | AMY99557.1 |
| *even-skipped* | *Priapulus caudatus* | AKU77019.1 |
| *even-skipped* | *Terebratalia transversa* | AHY88463.1 |
| *Mox* | *Dreissena rostriformis c2* | GHRL01028895 |
| *Mox* | *Dreissena rostriformis c1* | GHRL01028894.1 |
| *Mox* | *Acanthochitona crinita (fascicularis)* | GJJB01021710.1 |
| *Mox* | *Alitta virens* | AOS87316.1 |
| *Mox* | *Haliotis rufescens* | CAA53027.1 |
| *Mox* | *Mizuhopecten yessoensis* | XP_021347798.1 |
| *Mox* | *Terebratalia transversa* | AJV21315.1 |
| *Mox* | *Mus musculus* | CAA78812.1 |
| *Hox1* | *Acanthochitona crinita (fascicularis)* | APD15641.1 |
| *Hox1* | *Gymnomenia pellucida* | APD15663.1 |
| *Hox1* | *Nucula tumidula* | APD15698.1 |
| *Hox2* | *Acanthochitona crinita (fascicularis)* | AKV16303.1 |
| *Hox2* | *Gymnomenia pellucida* | APD15664.1 |
| *Hox2* | *Nucula tumidula* | APD15699.1 |
| *Hox3* | *Acanthochitona crinita (fascicularis)* | APD15643.1 |
| *Hox3* | *Nucula tumidula* | APD15700.1 |
| *Hox3* | *Wirenia argentea* | APD15711.1 |
| *Hox4* | *Dreissena rostriformis* | GHRL01028245 |
| *Hox4* | *Acanthochitona crinita (fascicularis)* | APD15644.1 |
| *Hox4* | *Nucula tumidula* | APD15701.1 |
| *Hox4* | *Wirenia argentea* | APD15712.1 |
| *Hox5* | *Acanthochitona crinita (fascicularis)* | APD15645.1 |
| *Hox5* | *Antalis entails* | APD15655.1 |
| *Hox5* | *Gymnomenia pellucida* | APD15667.1 |
