# Supplementary material for: A mosaic of conserved and novel modes of gene expression and morphogenesis in mesoderm and muscle formation of a larval bivalve
Source: Org Divers Evol. 2022 Jul 7;22(4):893–913. doi: 10.1007/s13127-022-00569-5 (PMC9649484; doi:10.1007/s13127-022-00569-5)
Supplement: Supplementary file 7 — Supplementary file7 (DOCX 16 kb) [file 13127_2022_569_MOESM7_ESM.docx]

| **Gene** | **species name (same in tree)** | **NCBI accession number / Ensembl Metazoa accession number*** |
| --- | --- | --- |
| *Brachyury* | *Dreissena rostriformis* | GHRL01029225 |
| *Brachyury* | *Acanthochitona crinita (fascicularis)* | GJJB01007210.1 |
| *Brachyury* | *Saccostrea kegaki* | BAG68616.1 |
| *Brachyury* | *Crassostrea gigas* | XP_011443609.1 |
| *Brachyury* | *Nematostella vectensis* | AAO27886.2 |
| *Brachyury* | *Patella vulgate* | CAD12821.1 |
| *Brachyury* | *Platynereis dumerilii* | CAC19335.1 |
| *Brachyury* | *Pomacea canaliculate* | XP_025098368.1 |
| *Brachyury* | *Tribolium castaneum* | NP_001034532.1 |
| *Brachyury* | *Lingula anatine* | XP_013385442.1 |
| *Eomes* | *Dreissena rostriformis* | GHRL01021264 |
| *Eomes* | *Cervus elaphus hippelaphus* | OWK02840.1 |
| *Eomes* | *Danio rerio* | NP_571754.3 |
| *Eomes* | *Xenopus tropicalis* | AAI67292.1 |
| *Tbx1* | *Nematostella vectensis* | AAQ23383.1 |
| *Tbx1* | *Crassostrea gigas* | XP_011442237.1 |
| *Tbx1* | *Mizuhopecten yessoensis* | OWF41599.1 |
| *Tbx2* | *Dreissena rostriformis* | GHRL01022900 |
| *Tbx2* | *Culex quinquefasciatus* | EDS41352.1 |
| *Tbx2* | *Crassostrea gigas* | * EKC26468.1 |
| *Tbx2* | *Mizuhopecten yessoensis* | XP_021365993.1 |
| *Tbx2* | *Lingula anatine* | XP_013379452.1 |
| *Tbx3* | *Dreissena rostriformis* | GHRL01036745.1 |
| *Tbx3* | *Lingula anatine* | XP_013379419.1 |
| *Tbx3* | *Biomphalaria glabrata* | XP_013085613.1 |
| *Tbx3* | *Crassostrea gigas* | XP_011436735.1 |
| *Tbx4* | *Cervus elaphus hippelaphus* | OWK13929.1 |
| *Tbx4* | *Larimichthys crocea* | XP_019124927.1 |
| *Tbx4* | *Tachysurus fulvidraco* | XP_027003995.1 |
| *Tbx5* | *Danio rerio* | AAF06733.1 |
| *Tbx5* | *Struthio camelus* | AIA82401.1 |
| *Tbx5* | *Cervus elaphus hippelaphus* | OWK14983.1 |
| *Tbx6* | *Danio rerio* | NP_705952.2 |
| *Tbx6* | *Xenopus laevis* | BAC20262.1 |
| *Tbx6* | *Mus musculus* | AAT72924.1 |
| *Tbx8* | *Toxocara canis* | KHN81917.1 |
| *Tbx8* | *Caenorhabditis elegans* | BAD14918.1 |
| *Tbx8* | *Pristionchus pacificus* | PDM70138.1 |
| *Tbx15* | *Dreissena rostriformis* | GHRL01011340.1 |
| *Tbx15* | *Mizuhopecten yessoensis* | OWF43000.1 |
| *Tbx15* | *Crassostrea gigas* | * EKC21660.1 |
| *Tbx15* | *Rattus norvegicus* | NP_001099921.1 |
| *Tbx15* | *Nestor notabilis* | KFQ43989.1 |
| *Tbx20* | *Dreissena rostriformis c1* | GHRL01025755 |
| *Tbx20* | *Dreissena rostriformis c2* | GHRL01025754 |
| *Tbx20* | *Mizuhopecten yessoensis* | OWF44695.1 |
| *Tbx20* | *Crassostrea gigas* | * EKC38760.1 |
| *Tbx20* | *Lingula anatine* | XP_013390981.1 |
| *Tbx20* | *Exaiptasia pallida* | KXJ24557.1 |
| *Tbx20* | *Cricetulus griseus* | RLQ68618.1 |
