# Supplementary material for: A mosaic of conserved and novel modes of gene expression and morphogenesis in mesoderm and muscle formation of a larval bivalve
Source: Org Divers Evol. 2022 Jul 7;22(4):893–913. doi: 10.1007/s13127-022-00569-5 (PMC9649484; doi:10.1007/s13127-022-00569-5)
Supplement: Supplementary file 8 — Supplementary file8 (DOCX 13 kb) [file 13127_2022_569_MOESM8_ESM.docx]

| **gene of interest** | **forward primer** | **reverse primer** | **insert length** |
| --- | --- | --- | --- |
| *Brachyury* | CCAAGTTCAAGGAATACACC | GCACGAAACTAGAGATGTC | 1037 bp |
| *even-skipped* | GATATGTATGAGGACGAC | GTGGAGTATTTATCGACTG | 849 bp |
| *Mox_c2* | GATCATAATCAGATGTACGGG | CCGACGAATAATCATCAC | 891 bp |
| *myosin II heavy chain_c1* | GATCCAGAGAAACATCAGG | GACTCATGCTGCATGGTC | 1122 bp |
