# Supplementary material for: A mosaic of conserved and novel modes of gene expression and morphogenesis in mesoderm and muscle formation of a larval bivalve
Source: Org Divers Evol. 2022 Jul 7;22(4):893–913. doi: 10.1007/s13127-022-00569-5 (PMC9649484; doi:10.1007/s13127-022-00569-5)
Supplement: Supplementary file 9 — Supplementary file9 (DOCX 15 kb) [file 13127_2022_569_MOESM9_ESM.docx]

|  | ***Brachyury*** | ***even-skipped*** | ***Mox_c1*** | ***Mox_c2*** | ***mhc_c1*** | ***mhc_c2*** | ***mhc_c3*** | ***mhc_c4*** |
| --- | --- | --- | --- | --- | --- | --- | --- | --- |
| **0 hpf** | 0,00 | 0,00 | 0,00 | 0,00 | 0,85 | 0,06 | 0,30 | 17,46 |
| **2 hpf** | 0,00 | 0,00 | 0,00 | 0,00 | 1,18 | 0,02 | 0,72 | 31,04 |
| **4 hpf** | 4,90 | 5,84 | 0,00 | 0,16 | 0,91 | 0,11 | 1,08 | 27,11 |
| **6 hpf** | 3,09 | 1,93 | 0,00 | 0,00 | 1,97 | 0,30 | 1,44 | 24,60 |
| **8 hpf** | 133,43 | 24,36 | 0,00 | 0,00 | 2,03 | 0,43 | 4,23 | 15,67 |
| **13 hpf** | 179,17 | 275,63 | 0,00 | 0,20 | 8,26 | 0,89 | 25,53 | 9,90 |
| **18 hpf** | 92,97 | 149,70 | 0,00 | 0,20 | 15,36 | 0,42 | 62,54 | 22,93 |
| **23 hpf** | 59,27 | 48,39 | 0,16 | 2,49 | 8,65 | 4,28 | 88,26 | 25,38 |
| **26 hpf** | 61,57 | 57,15 | 0,00 | 12,95 | 11,53 | 17,97 | 209,04 | 28,06 |
| **27 hpf** | 65,40 | 30,59 | 0,16 | 4,89 | 44,65 | 41,57 | 221,31 | 33,27 |
| **30 hpf** | 47,61 | 16,77 | 0,00 | 1,17 | 43,08 | 26,49 | 149,64 | 40,49 |
| **36 hpf** | 72,74 | 32,86 | 1,64 | 18,93 | 218,16 | 43,88 | 810,44 | 66,85 |
| **48 hpf** | 50,31 | 10,02 | 0,11 | 4,60 | 93,24 | 14,39 | 260,52 | 65,71 |
| **54 hpf** | 48,16 | 10,18 | 0,11 | 4,73 | 59,65 | 12,77 | 219,20 | 71,51 |
| **60 hpf** | 54,98 | 22,21 | 2,91 | 6,99 | 209,68 | 22,68 | 761,55 | 79,14 |
| **72 hpf** | 66,37 | 23,90 | 3,46 | 3,90 | 213,05 | 26,08 | 734,96 | 57,61 |
| **84 hpf** | 62,13 | 20,33 | 2,70 | 7,27 | 171,93 | 20,08 | 591,15 | 49,18 |
