# Supplementary material for: A mosaic of conserved and novel modes of gene expression and morphogenesis in mesoderm and muscle formation of a larval bivalve
Source: Org Divers Evol. 2022 Jul 7;22(4):893–913. doi: 10.1007/s13127-022-00569-5 (PMC9649484; doi:10.1007/s13127-022-00569-5)
Supplement: Supplementary file 10 — Supplementary file10 (DOCX 14 kb) [file 13127_2022_569_MOESM10_ESM.docx]

| **Gene** | **Nucleotide sequence** |
| --- | --- |
| *Dro-Bra* | CCAAGTCAAGGAATACACCAACGAGATGATTGTCACCAAAAACGGAAGGCGCATGTTCCCGGTGTTCAAGGCGAGCATCAGCGGCCTCGACCCCAACGCCATGTACTCGATCCTGCTCGACTTCGTCCAGGTGGACGCACACCGCTGGAAGTACGTGAACGGCGAGTGGGTCGCCGGCGGCAAGGCGGAACCCAGCGTGCCGAACTGCGTGTACGTACATCCGGACAGCCCGAACTTCGGCAGCCACTGGATGAAGGAGGCCATCTCGTTCTCCAAGGTCAAACTGACCAATAAGATGAACGGCGGAGGGCAGATCATGCTCAACTCGCTGCACAAGTATGAGCCCCGGCTTCACGTTGTCAAGGTGACGACGAACGCACAGAAGAAGCGGCTCAGCAGCTTCAACTTCCCGGGGACGCAGTTTATAGCGGTCACCGCGTACCAGAACGAAGAGATAACTGCATTGAAGATCAAACATAACCCGTTTGCAAAGGCTTTCCTGGACGCCAAAGAAAGACCGGAACAGAGAGAATTTTTAGAAGACAACTTGGACAACCAACAACGCTCTCTATCACATTTGACGGGGACTTGGTACATGCCCCCTGGTGGAGGTTTATTACCGCCACCCGCCCATCAGTTCGCCAACACCCTCGGCCTCGCCAACCCACACTGCGACCGCCTCTCCTTCCGGAACCCCCGGCACGCACCATATCCGCACCCCTACCAACGGCGCTCACCTCCGAACACGATATCCCGGGACATGAGTCAGAACCTGCCGATGTTGAACATCGCCGACAACTGGAGCCAGTTCACCAGCACCGCCGGCATGCTTAACTGTAACTCTGGCTCCCAGCCCCAGTACGGCATGTGGATGGGCTCCCATATGCCGGGAAACATTTCACCCAATCAGAACTGTTCTATGCCGTATCTGCGCAATGCCTATAACTCGATCCCGTCATCCACCACTAGTCAAAGTCACGTGACATCCTCTTCCGGTCTGATGTCTTTCGACCAGTGTGACATCTCTAGTTTCGTGC |
| *Dro-eve* | GAATTCGATTTATGTATGAGGACGACGGCGTGAGAGGGTCCATGATGTTCAAAGACGGCATGTTGGACGAGAACGGGATCCGGCGCTACAGAACAGCGTTCTCGCGAGAACAGATCGGTCGCCTCGAAAAGGAATTCTTCAAGGAGAACTATGTATCCCGGCCTCGTCGCTGCGAGCTCGCACAGGAATTGAACCTGCCGGAAAACACTATCAAGGTTTGGTTTCAAAACCGACGTATGAAAGACAAGCGACAGCGCATGGCAATGGCCTGGCCCCTGGGCAACGCTGACCCCCATTTGTACGCCTACCTCGCAGCTGCCGCTGCCTCCTACCCATACGGAATACCTCAGACCTCACCCATCAACTACTACAACACAATCGGACTCCAACGACCGACGCTACCTCAAACGATGCCTACGTCACCAGTGACGTCACAAGGCATCGGCGCCCTCGGACTTGGACAATATCCGTTTCCAAATCCGCTGCGTCCGCGTCAGGACCAGCTCCCCGGAATGTCGTCTGCGTTCTTCGGTGGACGATGTGCCACAATGCATCCCCAGCATCCGTTCCACAGTACGCCGTTGAACGGGAGTCACTTCCACCACGACGCCTCGCCACAGATGCTGAACACAAGTGGTGCTCTGAACTCAAGCGGTGGCTCGCTTTCGCCATCTTTAGAAGAAGCCGGTTTCCTGTCATCAACCGGAAACCCGTTGCTCAGTATGTCACTGAGCTCCAACAGAAAATCGCCGACCTCCGTTTCTCCCGTGTCTTCGGACATGAGTTCAAGAAAATCTCCCAGCGATGCGCGAGTGAAAGGCACCAATCCTCAGTCGATAAATACTCCAC |
| *Dro-Mox_c2* | TCCCGACGAATAATCATCACAATGTGCATCACTCCCTTCCACGGTGCGCATGTCCGCGCACTCCCGTTCGGCGTCCGACGTCACAACCGGCGTCATTGACGTCACTGGTTTGACCTGACCGGAGACCTTGTCCTTGACCACGTGAGTGCCCTTTACCCGTTTCCACTTCATGCGACGGTTCTGAAACCACACCTTGACCTGCCGTTCCGTAAGATCCAGTGCCACCGCGATCTCGTAACGTCGAAGTCTCGTGAGATAGTTGCGAGACGTGAACTCGCGTTCCAGCTCCCGGATCTGATGTTTCGTGAACGCCGTCCGCTCCTTACGGGGTTTGATAGAGAGATCGAGCTTGCATTGGTCGCCGGAATCGTCCAGCGTCTTATCGCTGTCGTCTGTTAGTCCATATTTCGTATCCGTGTCAATGTCGTCTGCAAATGTTGGACTGTGCAAACTCTGCCCAGCCTCGTTTTCCCGGCAATATCCATCTTTCAATGATGAAGAGTATTTCGAACTCTTAGTTCTTGGGTTACAAAAGTCACTATAATATCCGGCGGAATAATTATGTCCGATATCCGAACTTTTACTCCTGTCGTGTAATGATGATAACTGGCCGCTTTTTGACTCCGGWTGTACAGTCTCGCCGGCTTTTGATTCCGGTAGTAAGTAATTCGAATGATACGACGCCACGTTCCAGATTTGCGTGTACGACGGACCGTACTCCGAGAAAGGGTACGGCTGACAGGCGACCGAACGCGGGTCGTACGAGTGGGCATCGAACACGCCGGGCCGCGACAGACTGCCCGCCGGAAAGCCATAGCTGCCGCCGTGGAATGCGGACTGGAAATGATTTAAGTTGGATCCATTCACCCCGTACATCTGATTATGATCAAT |
| *Dro-mhc_c1* | GATCCAGRGAAACATCMGGAAATGGATGGTACTGAGGAACTGGCAGTGGKGGAAAATGTACACCAAGGTCAAGCCTCTCCTCAACGCTGCCCGCGCTGACGACGAGGTGAAGAAGATGGAAGAAGAGTTCGCCAAGACTAAGGAAGAGCTTGCAAAGGTCGAGAAGATCAAGAAGGAACTTGAAGAACGCTGCGTTAAGCTACAGAGGGAAAAGGAGGACGCTGTGCTTCAGCTTGCAGCTGAGGGCGATACCCTCGGTGATATGGAGGAAACCATTGAGAATCTCACCAAGCAGAAGTTCGAGTATGAAGCTCAGTTAAAGGAAATGGAAGATAGAATGTCTGAGGAGGAAGGAGAAGTTGGCAAGCTCGCTGCCCAAAAACAAGAACTTGAGGCTCAGGCTGCCAAGCTGAATGAGAACATCGCAGGTCTTGAAGATTCACTCAAGAAGGCTGAACACGACCGTCAGGTGAAGGACAACCAGATCAAGACACTGCAGGGAGAGATGGCCCAGCAGGACGAGCAGATTGGCAAGCTGACGAAGGAGAAGAAGGCGCTTGACGACACCGTCAAGAAGACCCAGGACGCTCTGAAGGCTGAGGAAGAAAAGGTGAACAACCTCAACAAGCTCAAGCAGAAGTTGGAGAGCACTCTTGATGAGCTTGAGGACAATCTTGAACGCGAGAAGAAGATTCGTGCTGATGTAGAGAAGGCCAAGAAGAAGTTGGAAACAGACTTGGCTGACACTCAGTCCGCTGTCGAGGAATTGGCACGCATCAAGTCGGATCTTGAGGGCGGCATCAAGAAAAAGGAGACTGAGCTCAAGAACCTGAGCCAGAAACTGGAAGAGGAGCAGGGTCTTGTTGCTCAACTCCAGAAAAAGATCAGGGATCTGCAGGCCCGCATCGAAGAACTCGAAGAAGAGCTCGAGGCTGAAAGGCAGGCCAGGGCTAAGGCCGAGAAAGCGAAGAACGACCTCGCCCGGGAACTGGAAGAATTAGCCGAGGCCCTTGATGAGCAGGGAGGTCAGACTGCCGCCCAGGTTGAGCTAAACAAAAAGCGAGAAGCCGAGGTGCAGAAGCTGCGTCGCGATCTGGAGGAACAGACCATGCAGCATGAGTC |
