# Supplementary material for: A mosaic of conserved and novel modes of gene expression and morphogenesis in mesoderm and muscle formation of a larval bivalve
Source: Org Divers Evol. 2022 Jul 7;22(4):893–913. doi: 10.1007/s13127-022-00569-5 (PMC9649484; doi:10.1007/s13127-022-00569-5)
Supplement: Supplementary file 11 — Supplementary file11 (DOCX 14 kb) [file 13127_2022_569_MOESM11_ESM.docx]

| **annotated gene** | **Domains** | **NCBI accession number** |
| --- | --- | --- |
| *Dreissena_rostriformis_Brachyury* | T-box | GHRL01029225 |
| *Dreissena_rostriformis_Eomes* | fragmented T-box | GHRL01021264 |
| *Dreissena_rostriformis_Tbx2* | T-box | GHRL01022900 |
| *Dreissena_rostriformis_Tbx3* | T-box | GHRL01036745.1 |
| *Dreissena_rostriformis_Tbx15* | T-box | GHRL01011340.1 |
| *Dreissena_rostriformis_c1_Tbx20* | T-box | GHRL01025755 |
| *Dreissena_rostriformis_c2_Tbx20* | T-box | GHRL01025754 |
| *Dreissena_rostriformis_even-skipped* | Homeodomain | GHRL01002417 |
| *Dreissena_rostriformis_c1_Mox* | Homeodomain | GHRL01028894.1 |
| *Dreissena_rostriformis_c2_Mox* | Homeodomain | GHRL01028895 |
| *Dreissena_rostriformis_Hox4* | Homeodomain | GHRL01028245 |
| *Dreissena_rostriformis_c1_myosin_I* | fragmented myosin head, myosin TH1 | GHRL01034922 |
| *Dreissena_rostriformis_c2_myosin_I* | fragmented myosin head, 2x IQ | GHRL01003178 |
| *Dreissena_rostriformis_c1_myosin_II*  *_heavy_chain* | myosin N, myosin head, myosin tail 1 | GHRL01011381 |
| *Dreissena_rostriformis_c2_myosin_II*  *_heavy_chain* | myosin N, myosin head, IQ, myosin tail 1 | GHRL01034471 |
| *Dreissena_rostriformis_c3_myosin_II*  *_heavy_chain* | myosin N, myosin head, myosin tail 1 | GHRL01017649 |
| *Dreissena_rostriformis_c4_myosin_II*  *_heavy_chain* | fragmented myosin head, myosin tail 1, myosin tail 1 | GHRL01006544 |
| *Dreissena_rostriformis_myosin_III* | Pkinase, myosin head, 5x IQ | GHRL01023082 |
| *Dreissena_rostriformis_myosin_V* | fragmented myosin head | GHRL01001522 |
| *Dreissena_rostriformis_myosin_VI* | myosin N, fragmented myosin head, myosin VI – CBD | GHRL01023773 |
| *Dreissena_rostriformis_myosin_VII* | myosin head, 3x IQ | GHRL01032474 |
| *Dreissena_rostriformis_myosin_IX* | 3x fragmented myosin head, 3x IQ, fragmented RhoGAP | GHRL01016224 |
| *Dreissena_rostriformis_myosin_XV* | 3x fragmented myosin head, MyTH4 | GHRL01005003 |
| *Dreissena_rostriformis_myosin_XVIII* | fragmented myosin head | GHRL01021375 |
